# Composite antiferromagnetic and orbital order with altermagnetic properties at a cuprate/manganite interface

**DOI:** 10.1093/pnasnexus/pgae100

**Published:** 2024-03-04

**Authors:** Subhrangsu Sarkar, Roxana Capu, Yurii G Pashkevich, Jonas Knobel, Marli R Cantarino, Abhishek Nag, Kurt Kummer, Davide Betto, Roberto Sant, Christopher W Nicholson, Jarji Khmaladze, Ke-Jin Zhou, Nicholas B Brookes, Claude Monney, Christian Bernhard

**Affiliations:** Department of Physics and Fribourg Center for Nanomaterials, University of Fribourg, Fribourg CH-1700, Switzerland; Department of Physics, West University of Timisoara, Timisoara 300223, Romania; Department of Physics and Fribourg Center for Nanomaterials, University of Fribourg, Fribourg CH-1700, Switzerland; O. Galkin Donetsk Institute for Physics and Engineering NAS of Ukraine, Kyiv 03028, Ukraine; Department of Physics and Fribourg Center for Nanomaterials, University of Fribourg, Fribourg CH-1700, Switzerland; Department of Physics and Fribourg Center for Nanomaterials, University of Fribourg, Fribourg CH-1700, Switzerland; European Synchrotron Radiation Facility, F-38043 Grenoble Cedex 9, France; Diamond Light Source, Harwell Campus, Didcot, Oxfordshire OX11 0DE, UK; European Synchrotron Radiation Facility, F-38043 Grenoble Cedex 9, France; European Synchrotron Radiation Facility, F-38043 Grenoble Cedex 9, France; European Synchrotron Radiation Facility, F-38043 Grenoble Cedex 9, France; Department of Physics and Fribourg Center for Nanomaterials, University of Fribourg, Fribourg CH-1700, Switzerland; Department of Physics and Fribourg Center for Nanomaterials, University of Fribourg, Fribourg CH-1700, Switzerland; Diamond Light Source, Harwell Campus, Didcot, Oxfordshire OX11 0DE, UK; European Synchrotron Radiation Facility, F-38043 Grenoble Cedex 9, France; Department of Physics and Fribourg Center for Nanomaterials, University of Fribourg, Fribourg CH-1700, Switzerland; Department of Physics and Fribourg Center for Nanomaterials, University of Fribourg, Fribourg CH-1700, Switzerland

**Keywords:** superconductivity, magnons, altermagnetism, cuprates, RIXS

## Abstract

Heterostructures from complex oxides allow one to combine various electronic and magnetic orders as to induce new quantum states. A prominent example is the coupling between superconducting and magnetic orders in multilayers from high-$T_{c}$ cuprates and manganites. A key role is played here by the interfacial CuO_2_ layer whose distinct properties remain to be fully understood. Here, we study with resonant inelastic X-ray scattering the magnon excitations of this interfacial CuO_2_ layer. In particular, we show that the underlying antiferromagnetic exchange interaction at the interface is strongly suppressed to J≈70 meV, when compared with J≈130 meV for the CuO_2_ layers away from the interface. Moreover, we observe an anomalous momentum dependence of the intensity of the interfacial magnon mode and show that it suggests that the antiferromagnetic order is accompanied by a particular kind of orbital order that yields a so-called altermagnetic state. Such a 2D altermagnet has recently been predicted to enable new spintronic applications and superconducting proximity effects.

**Significance Statement**

We report a resonant inelastic X-ray scattering study of multilayers made from a cuprate high-$T_{c}$ superconductor and a magnetic perovskite manganite. Our study reveals an extraordinary behavior of the spins and electrons of the interfacial cuprate monolayer. In particular, we observe that its antiferromagnetic spin interaction is strongly suppressed and we make the fundamental discovery that it hosts a new kind of combined magnetic and electronic order that constitutes a 2D altermagnetic state. Our findings significantly advance the state of the art in the field of altermagnets that are of great current interest since they enable new kinds of spintronic and magnonic devices and may also lead to exotic superconducting proximity effects.

## Introduction

The proximity effect at the interfaces between different materials with strongly correlated electrons, such as Mott-type antiferromagnetic (AF) insulators, cuprate high-$T_{c}$ superconductors, or the colossal-magneto-resistance manganites, is of great current interest in fundamental and applied sciences. Specifically, the cuprate/manganite interface holds great promises for inducing unconventional quantum states which have potential applications, e.g. in spintronics or quantum computation.

This creates an urgent need for experimental techniques which can selectively probe the electronic and magnetic properties in the vicinity of the interfaces of such heterostructures. In the following, we demonstrate for the case of a cuprate/manganite interface that resonant inelastic X-ray scattering (RIXS) can provide unique information about the antiferromagnetic exchange interaction and the orbital order of the holes on the interfacial CuO_2_ layer.

The parent compounds of the high-$T_{c}$ cuprates are charge transfer insulators with a long-range AF order of the spins of the holes that reside on half-filled ${\mathrm{Cu-}3d}_{x^{2}-y^{2}}$ levels. Upon doping away from half-filling (the additional holes have a strong oxygen character and form so-called Zhang–Rice singlets (1)), the long-range AF order is rapidly suppressed and a strongly correlated conducting and eventually superconducting (SC) state develops. Static but short-ranged AF correlations persist to a higher hole doping and coexist with the SC order in parts of the so-called underdoped regime where $T_{c}$ increases with doping and eventually reaches a maximum around optimal doping.

Fluctuating AF correlations persist even beyond optimum doping (2) into the so-called overdoped regime where SC is suppressed and eventually vanishes. It is therefore widely assumed that the AF fluctuations are involved in the SC pairing (3). However, a consensus on this issue has not yet been reached, since the strong electronic correlations also give rise to a short-ranged charge order (4–7), and possibly even quadrupolar or octopolar orders (8) or so-called flux-phases (9), that may also coexist with high-$T_{c}$ superconductivity (10).

The properties of the AF spin fluctuations and their evolution upon hole doping have been extensively investigated with inelastic neutron scattering (INS). The studied materials range from La_2_CuO_4_ (11–13) to YBa_2_Cu_3_O_6+*δ*_ (13–16), La_2−*x*_(Ba,Sr)_*x*_CuO_4_ (13, 17), and other cuprates like Bi-2212 and Hg-1201 (18).

The INS studies have shown that the AF exchange interaction is strongly anisotropic, i.e. for YBCO the in-plane interaction amounts to $J_{∥}\approx 120-130$ meV, whereas the out-of-plane one is $J_{⊥}\approx 9-13$ meV for the coupling between the closely spaced CuO_2_ layers and $J_{⊥}^{\prime }\approx 0.02-0.4$ meV (14, 19) between the CuO_2_ bilayers units (across the CuO chains). The spin waves in the high-$T_{c}$ cuprates are therefore quasi-2D and disperse over an energy range of about 300 meV (16).

More recently, RIXS has emerged as another powerful technique to study the spin-wave excitations of the high-$T_{c}$ cuprates (20). The much larger interaction cross-section of this photon-based technique enables RIXS studies of the magnetic excitations on small single crystals and even on thin films. The RIXS technique also provides the unique possibility to probe the spin excitations (magnons) in an element-specific manner, which is especially helpful for the study of materials which contain different magnetic ions. The reported dispersion of the magnons is quite consistent with that previously reported from INS experiments. In particular, for underdoped YBa_2_Cu_3_O_6.6_ and YBa_2_Cu_4_O_8_, and even for optimally doped and slightly overdoped Nd_1.2_Ba_1.8_Cu_3_O_7_ and YBa_2_Cu_3_O_7_ cuprates, the RIXS studies have confirmed the persistence of paramagnon excitations due to slowly fluctuating and short-ranged AF spin correlations (21). Notably, an RIXS study of the thickness dependence of the magnon excitations in near optimally-doped NdBa_2_Cu_3_O_7_ thin films confirmed that the generic behavior of the magnons is similar to that in bulk samples. Remarkably, this holds even for a single unit cell film which was found to exhibit only a moderate reduction of the exchange interaction to $J_{∥}\approx 98$ meV (from a bulk-like value of 114 meV of the thicker films) (22).

In the following, we report a corresponding RIXS study of the magnetic excitations in a cuprate/manganite superlattice with 10 repetitions of 10 nm Nd_0.65_(Ca_0.7_Sr_0.3_)_0.35_MnO_3_ (NCSMO) and 7 nm YBa_2_Cu_3_O_7_ (YBCO) and a final 10 nm NCSMO cap layer. Bulk YBa_2_Cu_3_O_7_ is nearly optimally-doped high-$T_{c}$ superconductor with $T_{c}\approx 90$ K and NCSMO an insulator with a CE-type AF and charge orbital order (COO) that coexists and competes with a ferromagnetic order that is strengthened and prevails in large external magnetic fields (23). The resistance curves of the superlattice (NY-SL) show an onset of the SC transition around $T_{c}\approx 90$ K and the magnetization data reveal a weak ferromagnetic signal below about 120 K (that is enhanced by a large magnetic field) (24).

Previous studies with X-ray absorption spectroscopy (XAS) on similar cuprate/manganite heterostructures with ferromagnetic La_2/3_Ca_1/3_MnO_3_ layers have demonstrated that their electronic and magnetic properties in the vicinity of the interfaces are strongly modified. X-ray linear dichroism (XLD) measurements established that the Cu-d electrons of the interfacial CuO_2_ layers undergo a so-called orbital reconstruction whereby about 50% of the holes are redistributed from the $d_{x^{2}-y^{2}}$ to the $d_{3z^{2}-r^{2}}$ orbitals (25–31). They also revealed a transfer of electrons from the manganite to the cuprate side of the interface that reduces the hole doping of the interfacial CuO_2_ layer which thus is expected to have a weakened SC order and host a static AF order. Moreover, X-ray and magnetic circular dichroism (XMCD) studies have identified an induced ferromagnetic Cu moment that is antiparallel to the Mn moment (32–34). The above-described phenomena have been phenomenologically described in terms of the hybridization between the ${\mathrm{Cu-}3d}_{3z^{2}-r^{2}}$ orbitals of the Cu and Mn ions via the Cu–O^ap^–Mn bonds (O^ap^: apical Oxygen) across the interface (25).

Previous RIXS studies of such cuprate/manganite multilayers have demonstrated that the Cu-charge density wave order and also the crystal field excitations (dd-excitation) of the interfacial CuO_2_ layers can be tuned via the hole doping (*x*) and the tolerance factor of the manganite layers (30). Moreover, a new kind of Cu-based charge order with a large wave length of about 10 YBCO unit cells, a sizeable coherence length of 40 nm, and a $d_{z^{2}}$ character rather than the usual $d_{x^{2}-y^{2}}$ one, has been observed in some of these multilayers (31).

Corresponding RIXS studies of the magnetic excitations in heterostructures from transition metal oxides are rare and do not have the energy resolution required for a fine-structure analysis as to distinguish between the contributions from the interface and from the central part of the layers (35). In particular, there exists, to our best knowledge, no corresponding RIXS study of the spin order at the cuprate/manganite interfaces. Moreover, little is known about a possibly related orbital order which may accompany the orbital reconstruction that yields similar amounts of holes with $d_{x^{2}-y^{2}}$ and $d_{3z^{2}-r^{2}}$ orbital character. Notably, such a combined AF and orbital order might give rise to a so-called altermagnetic state (36) that has recently obtained great attention since it yields a spin-splitting of the Fermi-surface which changes its sign along different directions and can be sizeable even without a strong spin-orbit coupling, thus being potentially useful for various spintronic or magnonic applications. Particularly interesting are devices from superconductors and altermagnets for which the proximity effect and the resulting Andreev reflection are predicted to be strongly dependent on the interface orientation (37).

## Results and discussion

### XAS study

**Fig. 1.**
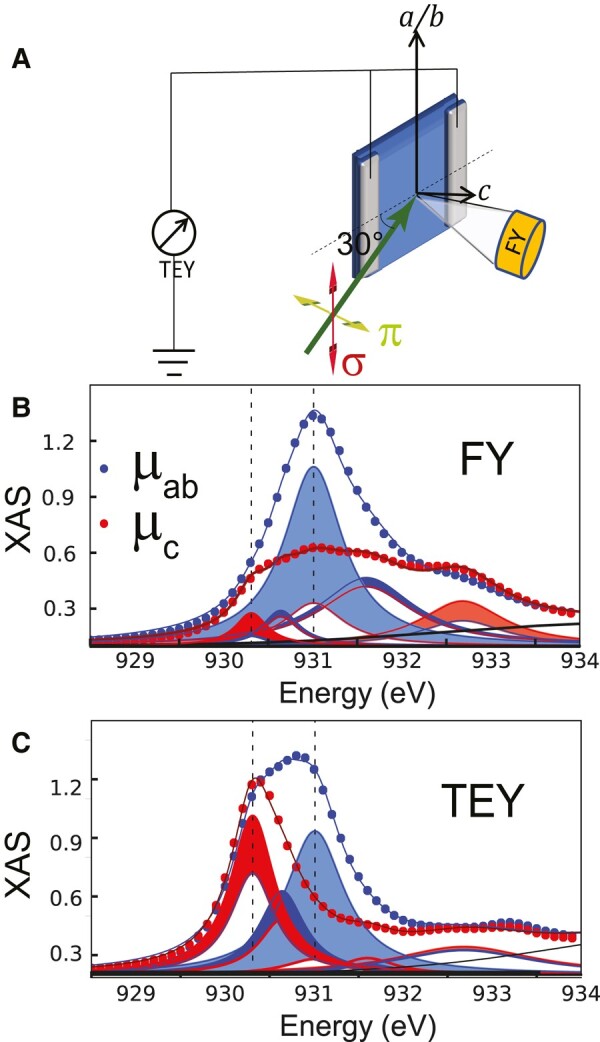
XAS data showing the charge transfer and orbital reconstruction effects of the interfacial CuO_2_ layer. A) Schematics of the XAS experiment in FY and TEY modes. B and C) FY and TEY spectra and multipeak fits for the in-plane and out-of-plane components of the linearly polarized incident X-rays. Blue (red) shaded areas denote contributions with ($\mu_{\mathrm{ab}}-\mu_{c})>0$ (<0), where $\mu_{\mathrm{ab}}$ ($\mu_{c}$) is the net absorption along the in-plane (out-of-plane) direction, as explained in Section S1.1. (40).

Figure 1 shows representative XAS spectra at the Cu-L_3_ edge which confirm that our NY-SL exhibits the same kind of interfacial charge transfer and orbital reconstruction effects as those that were previously reported for corresponding manganite/cuprate heterostructures (25–29, 31–33). Figure 1A shows a sketch of the XAS experiment with the X-rays in *π*- or *σ*-polarization incident at 30° with respect to the film plane. As indicated, the X-ray absorption spectra have been recorded simultaneously in fluorescence yield (FY) and total electron yield (TEY) modes at 4 K. Due to the small escape depth of the excited electrons, the TEY response is governed by the topmost cuprate layer, which, for our NY-SL is the interfacial CuO_2_ layer located underneath the final NCSMO layer. The FY signal is hardly depth sensitive, on the scale of the layer thickness of tens of nanometers, and thus represents the average response of the Cu ions throughout the SL. Figure 1B confirms that the latter FY spectra are similar to those reported for bulk YBCO (29, 38, 39). In particular, they exhibit a resonance peak at 931 eV, that is much stronger in ab-polarization than in c-polarization and thus is characteristic of Cu-3d^9^ holes that reside predominantly in the $d_{x^{2}-y^{2}}$ orbital. The corresponding TEY spectra in Fig. 1C reveal a remarkably different behavior that is characteristic of an interfacial charge transfer and an orbital reconstruction of the interfacial Cu ions. The charge transfer is evident from a substantial red-shift of the resonance peak of the interfacial Cu ions from 931 to about 930.4 eV. In the RIXS studies section, we will use this characteristic difference in the resonance energies of the bulk-like and the interfacial Cu ions to distinguish their respective magnon excitations as seen in the RIXS experiment. The orbital reconstruction is evident in the XAS spectra from a strong enhancement of the *c*-axis component of the 930.4 eV peak, for which the intensity is comparable to that of the ab-plane component, signaling that the holes in the interfacial CuO_2_ layer are more or less equally distributed between the $d_{x^{2}-y^{2}}$ and $d_{3z^{2}-r^{2}}$ orbitals.

### RIXS studies

**Fig. 2.**
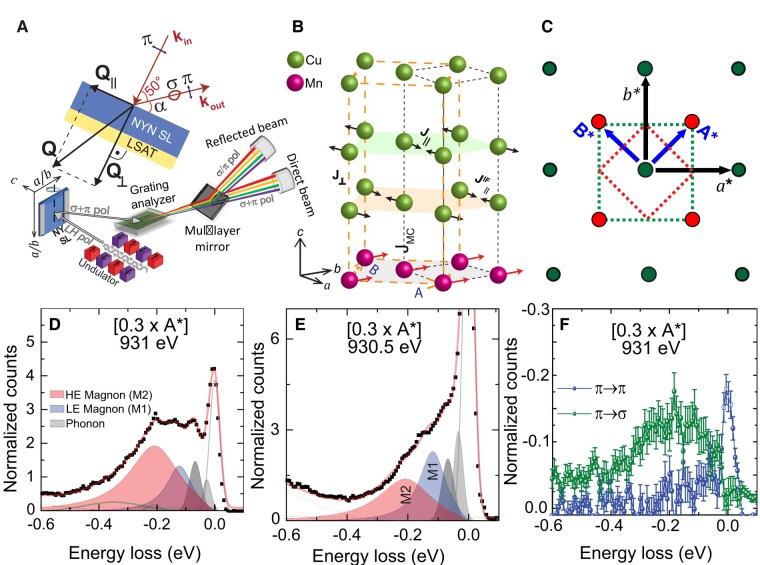
RIXS study of the distinct magnon modes of the bulk-like and interfacial CuO_2_ layers. A) Sketch of the RIXS and polarimetry experiments indicating the measurement geometry. B) Scheme of the magnetic order and exchange interaction of the Cu- and Mn-spins in the vicinity of the cuprate/manganite interface. C) Reciprocal lattice vectors of the structural (green dots) and the magnetic (red dots) orders with the corresponding first BZ shown by dotted lines. D and E) Representative RIXS spectra at [0.3×A*] showing the two magnon modes M1 (blue shading) and M2 (red shading) along with phonons (gray shading) and the elastic line (gray line) at the bulk-like Cu resonance of 931 eV and the interfacial Cu resonance of 930.5 eV, respectively. The spectra are normalized with respect to the area of the dd-excitations beyond −1 eV (not shown). F) Corresponding RIXS–polarimetry spectra at [0.3×A*] and 931 eV showing that the M1 and M2 modes are predominantly from spin-flip scattering and thus of magnetic origin. The error bars have been calculated as described in Section S3.2 (40).

Next, we turn to the high-resolution RIXS study at the Cu-L_3_ edge of the NY-SL that has been conducted at the I21 beamline of the Diamond light source at 20 K using a grazing exit geometry with *π*-polarization of the incident X-ray beam and a scattering angle of 50°, as sketched in Fig. 2A. The RIXS spectra have been measured with an energy resolution of 42 meV. They have been corrected for self-absorption effects and subsequently normalized to the area of the dd-excitations above 1 eV (as shown in Figs. S4 and S5 (40)). Figure 2B shows a sketch of the lattice structure of YBCO and NCSMO and of the magnetic structure of the Cu- and Mn-spins in the vicinity of the cuprate/manganite interface. The related structural (green dots) and magnetic (red dots) reciprocal lattice vectors of YBCO are displayed in Fig. 2C, where the dotted lines show the corresponding first Brillouin zones (BZ). Series of RIXS measurements have been performed by varying the sample rotation angle from $\alpha =9.8^{\circ }$ to $54.7^{\circ }$ (Fig. 2A) as to map out the dispersion along the in-plane momentum directions [h,0] and [h,h] with respect to the crystallographic BZ of YBCO. Here, we use the convention that the vectors [h,0] and [h,h] are written in units of a* and A*, respectively, as detailed in Fig. 2C. In agreement with previous RIXS studies (2, 21, 22, 41–47), we assume that the dispersion of the relevant orders and excitations along the out-of-plane direction is very weak and can therefore be neglected.

The crystal field (dd) excitations at higher energy agree rather well with those previously reported for bulk YBCO (44, 48) (see also Section S2.2 and Fig. S6 (40)). In the following, we focus on the low-energy region of the RIXS spectra (below −0.6 eV) which contains, besides the elastic peak, contributions from inelastic excitations, like phonons, magnons, and bimagnons. More details about the fitting of these low-energy features can be found in Section S2.2 (40).

Figure 2D shows a representative RIXS spectrum below −0.6 eV at the bulk Cu resonance energy of 931 eV and a momentum vector of 0.3A*. It contains two high-energy phonons around 60 and 80 meV (gray shading) that can be assigned to the buckling and breathing modes of YBCO, respectively (49). The phonon modes at lower energy are not resolved and thus contribute to the elastic peak (gray line). Following the analysis of a previous high-resolution RIXS study of NaBa_2_Cu_3_O_6_ (50), the fairly weak and broad peak around 350 meV (dark green line) is assigned to a bi-magnon excitation. Notably, the spectrum contains two strong, additional modes with maxima around 120 and 200 meV (blue and red shadings) which dominate the signal above the phonon range at ΔE>80 meV. In the following, we show that both modes are of magnetic origin, and, accordingly, we denote them as M1 and M2 modes. Moreover, we provide evidence that the M2 mode corresponds to the magnon of the bulk-like CuO_2_ layers that are located away from the interface whereas the M1 mode arises from the magnetic excitations of the interfacial CuO_2_ layers. This assignment is supported by the comparison of Fig. 2D and E, which show the RIXS spectra at the momentum transfer 0.3A* for the incident photon energies of 931 and 930.5 eV at the resonances of the bulk-like and the interfacial Cu ions, respectively (see the XAS spectra in Fig. 1). This comparison highlights that the M2 mode is the most pronounced feature in the spectrum at 931 eV (bulk resonance), whereas the M1 mode prevails at 930.5 eV (interfacial resonance).

To ascertain the magnetic nature of the M1 and M2 modes, we performed additional RIXS–polarimetry measurements at the ID32 beamline of ESRF (51) at 20 K. This experiment resolves the polarization of the incident and of the scattered X-rays. Accordingly, it allows one to distinguish between the nonspin flip (*π*–*π*) and the spin-flip (*π*–*σ*) signals of which only the latter arises from scattering with a single magnon. As shown schematically in Fig. 2A, the polarization analysis is done by inserting after the analyzer a mirror with different reflection coefficients for *σ*- and *π*-polarized X-rays. Figure 2F shows the obtained polarization resolved spectra at 0.3A*, after self-absorption correction and normalization. It confirms that the signal in the energy-loss range of the M1 and M2 peaks is governed by the spin-flip channel and is therefore predominantly due to single-magnon scattering. Except for the low-energy range with the elastic peak and the phonons (below about 100 meV), the nonspin-flip (*π*–*π*) signal in Fig. 2D is much lower than the spin-flip one. Only two weak peaks around 0.38 and 0.2 eV, that agree well with the bimagnons of the fits in Fig. 2D, rise here above the calculated error bar.

With this information at hand, we analyzed the full, angle-dependent series of RIXS spectra (without polarization analysis of the scattered beam) at the bulk-like resonance of 931 eV and near the interface resonance of 930.5 eV. Both series have been fitted simultaneously using the two magnon modes M1 and M2. Since the YBCO layers in our sample are optimally doped, the magnons are expected to be fairly broad and resemble the response function of an overdamped harmonic oscillator (52).

**Fig. 3.**
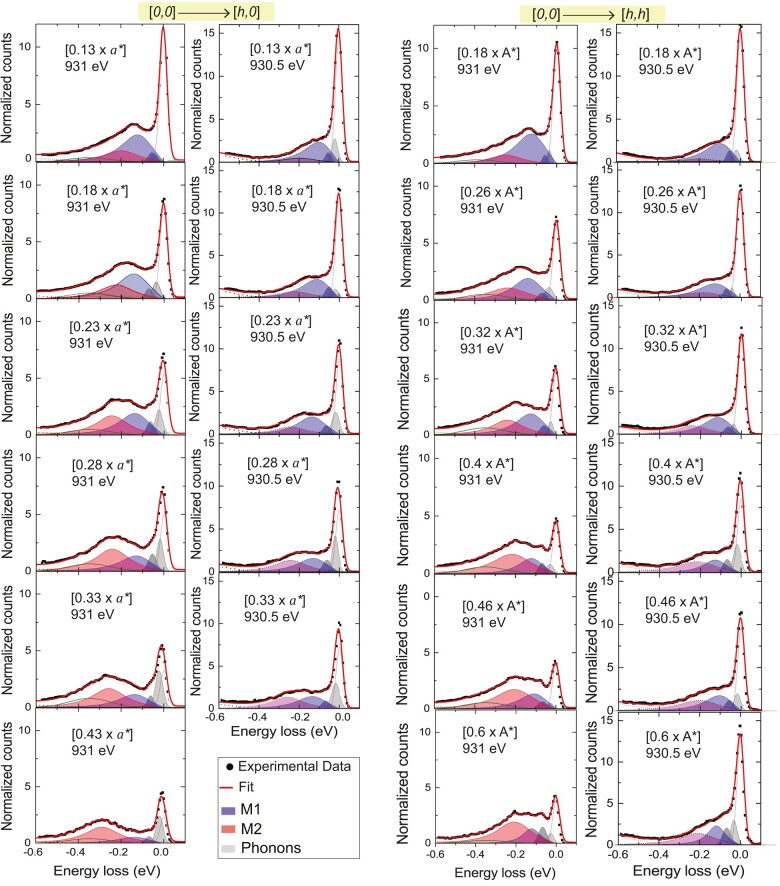
Comparison of the RIXS spectra (black dots) and their fitting (solid lines and color shading) at the bulk resonance energy of 931 eV and the interface resonance energy of 930.5 eV along [h,0] and [h,h]. The contributions of the M1 and M2 modes are shown by the blue and red shadings, respectively. The legend is presented at the bottom of the 2nd column.

**Fig. 4.**
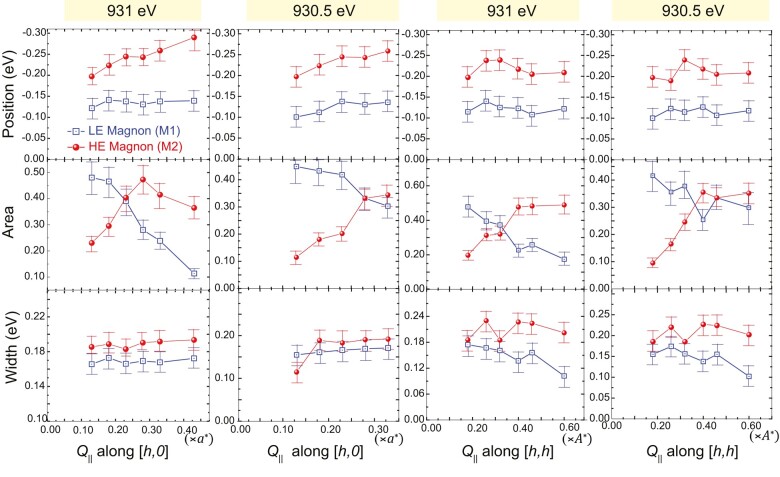
Summary of the best fit parameters obtained for the dispersion of the magnon modes M1 (blue squares) and M2 (red spheres) along [h,0] and [h,h] at the bulk resonance energy of 931 eV and the interface resonance energy of 930.5 eV, respectively. Calculation of the error bars is discussed in Section S2.3 (40).

The measured RIXS spectra and the corresponding fits are displayed in Fig. 3, and the dispersion of the best fit parameters of the magnon modes M1 (blue hollow squares) and M2 (red solid dots) is summarized in Fig. 4. The parameters of the M2 mode and their dispersion along the [h,0] and [h,h] directions are similar to those reported for bulk RBCO and corresponding thin films (2, 21, 43, 45). Specifically, the energy of the M2 mode has a maximal value of about 300 meV at the largest wave vector of 0.43a* and it decreases continuously toward smaller values of *h*. Moreover, the intensity of the M2 mode shows a strong decrease toward small *h* values similar to the bulk magnons in AF YBCO (2, 21, 43, 45, 49). To the contrary, the M1 mode has a much lower maximal energy of about 120 meV and is only weakly dispersive. Notably, the evolution of the intensity of the M1 mode is opposite to that of the M2 mode, i.e. it is weakest at large *h* values and increases strongly as *h* decreases. The distinct resonance energy, the weakly dispersive behavior, and the unusual spectral weight increase toward small in-plane momentum transfer thus clearly distinguish the M1 mode from the usual magnon excitations in YBCO or related planar high-$T_{c}$ cuprates (21, 49, 53).

## Comparison of RIXS data with linear spin-wave model

### Linear spin-wave model

In the following, we show that a minimal model based on linear AF spin-wave theory can account for the main features of the M1 and M2 modes, except for the unusual intensity increase of the M1 mode toward small wave vectors. The schematics of this minimal spin wave model is depicted in Fig. 2B. For the CuO_2_ layers that are not located right at the cuprate/manganite interface, we adopt a 2D Heisenberg nearest neighbor model with an in-plane AF exchange interaction, $J_{∥}$, and an interplanar AF exchange within the CuO_2_ bilayer units, $J_{⊥}\ll J_{∥}$, similar to that in bulk YBCO. For the interfacial CuO_2_ bilayer unit we assume that only the CuO_2_ layer that is directly bonded via an apical oxygen to the adjacent MnO_2_ layer has strongly modified electronic, orbital, and magnetic properties. Because the RIXS spectra can be fitted well with only two magnon modes, we assume that the second CuO_2_ plane of this bilayer unit has already bulk-like properties.

Finally, our minimal spin-wave model also includes a weak AF exchange coupling across the interface with the adjacent MnO_2_ layer, $J_{\mathrm{MC}}$. However, as detailed in Sections S1.3 and S4.1, it turns out that $J_{\mathrm{MC}}$ is very small, i.e. below 1 meV, and thus affects the magnon dispersion of the interfacial CuO_2_ layer only at very low energies that are not relevant for the analysis of the dispersion of the magnon data (M1 mode) that is presented in the following paragraph.

### Fitting of magnon dispersion

**Fig. 5.**
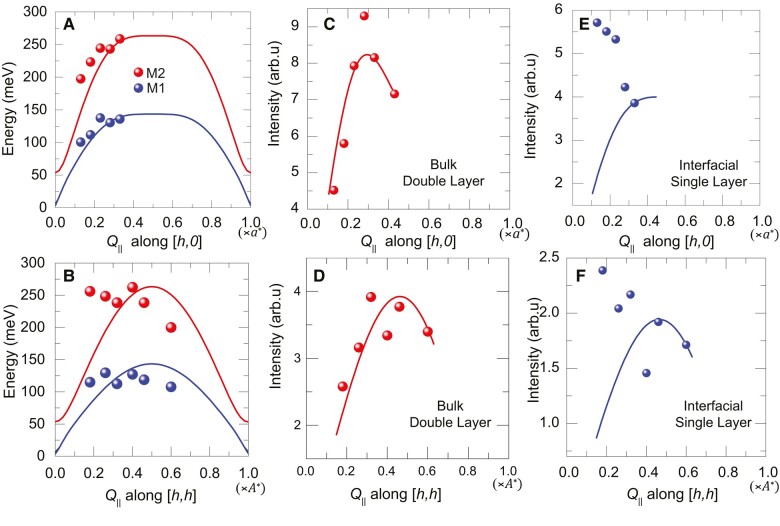
Fitting of the magnon dispersion in the bulk-like and interfacial CuO_2_ layers with a linear spin-wave model. A and B) Dispersion of the magnon energy along the (*π*,0) and (*π*,*π*) directions. C and D) Corresponding dispersion of the intensity of the bulk-like magnon and E and F) of the magnon of the interfacial CuO_2_ layer. Note that the calculated intensities have been scaled at the highest wave vector to those of the experimental data point.

Figure 5 shows a comparison of the dispersion of the M1 and M2 modes of the RIXS data with that of the best fits with the above-described minimal spin-wave model for which the M1 and M2 modes are assigned to the magnons of the interfacial and the bulk-like CuO_2_ layers, respectively. Figure 5A and B compare the dispersion of the magnon energy along the [h,0] and [h,h] directions, respectively.

For the fitting, only the in-plane exchange parameters ($J_{∥}$ and $J_{∥}^{\mathrm{IF}}$) were allowed to vary. To keep the number of fitting parameters at a minimum, we fixed the out-of-plane interaction of the bulk-like CuO_2_ layer to $J_{⊥}=7$ meV (as in bulk YBCO). Likewise, we used a fixed value of $J_{\mathrm{MC}}=0.5$ meV for the exchange coupling between the interfacial CuO_2_ and MnO_2_ layers. Note that both of these small out-of-plane exchange parameters do not have a noticeable effect on the magnon dispersion curves in the relevant energy range of the M1 and M2 modes above 100 meV.

The best fit of the in-plane exchange interactions yields values of $J_{∥}=130$ meV and $J_{∥}^{\mathrm{IF}}\approx 70$ meV. The former parameter, that determines the dispersion of the M2 mode in the bulk-like CuO_2_ layers, is indeed similar to that reported for bulk [Re]Ba_2_Cu_3_O_7_ or thin films of [Re]Ba_2_Cu_3_O_7_ (13, 16, 21, 22, 41). The latter parameter signals a strong suppression of the in-plane AF exchange coupling of the interfacial CuO_2_ layer. As discussed in Ref. (28), such a large suppression of $J_{∥}^{\mathrm{IF}}$ is expected from the orbital reconstruction of the interfacial CuO_2_ layer, which increases the population of holes on the ${\mathrm{Cu-}3d}_{3z^{2}-r^{2}}$ orbitals and thus gives rise to Cu($d_{3z^{2}-r^{2}}$)−O−Cu($d_{x^{2}-y^{2}}$) and Cu($d_{3z^{2}-r^{2}}$) −O−Cu($d_{3z^{2}-r^{2}}$) bonds that have a lower hopping probability than the Cu($d_{x^{2}-y^{2}}$) −O−Cu($d_{x^{2}-y^{2}}$) bonds that prevail in the bulk-like CuO_2_ layers.

Figure 5C, D and E, F displays the comparison of the measured (symbols) and calculated (lines) dispersion of the intensities of the magnon modes for the bulk-like and the interfacial CuO_2_ layers, respectively. Since the absolute values of the experimental intensities cannot be quantified, the comparison with the theoretical values has been facilitated by rescaling them so they are matched at the largest wave vector of the measurement. For the M2 mode from the bulk-like CuO_2_ layers, this yields a fairly good agreement between the experimental and the calculated magnon intensities. To the contrary, for the M1 mode there exists a striking discrepancy between the measured and the calculated magnon intensities. Whereas the linear spin-wave model predicts that the magnon intensity should decrease and finally vanish toward [0,0], the experimental magnon intensity exhibits a steep increase toward small wave vectors. This striking discrepancy reveals that our minimal spin-wave model is lacking an important feature of the magnetic and electronic state of the interfacial CuO_2_ layer. In the next paragraph, we show that the missing feature turns out to be an additional electronic order that is coupled in a very specific way with the antiferromagnetic order of the interfacial CuO_2_ layer.

### Combined AF and orbital order of the interfacial CuO_2_ layer

In the following, we show that the above-described discrepancy between the measured evolution of the intensity of M1 mode and that predicted by the minimal spin-wave model can be readily resolved in terms of an additional spatial order that develops concurrently with the AF order in the interfacial CuO_2_ layer. To account for the intensity increase of the M1 mode toward small wave vectors, this additional order needs to give rise to a strong modification of the amplitudes of the RIXS matrix elements for the spin-up and spin-down components of the AF state. As outlined in Section S4.3 and Eqs. S4.16 and S4.21 (40), this allows to remove the destructive interference effect on the RIXS intensity that occurs in the plain AF state for which the spin-up and spin-down components develop a $180^{\circ }$-phase shift at small scattering wave vectors.

**Fig. 6.**
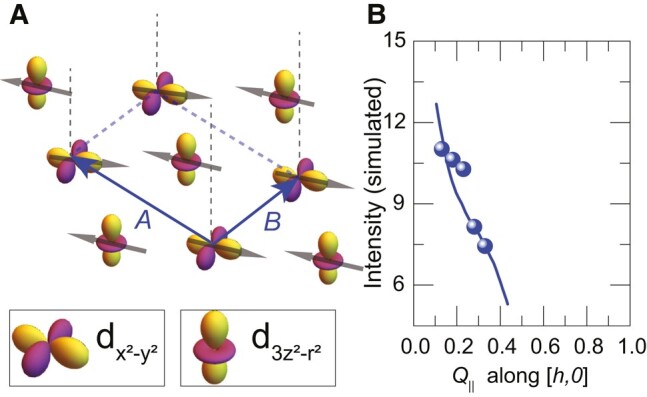
Evidence for a combined magnetic and orbital order at the interfacial CuO_2_ layer. A) Schematic of the proposed combined AF and checkerboard orbital order at the interfacial CuO_2_ layer. B) Comparison of the dispersion along [h,0] of the intensity of the M1 mode (dots) with that calculated for the model shown in panel A.

A natural candidate for causing the required modification of the scattering amplitudes of the spin-up and spin-down components of the AF state is a lateral order of the $d_{x^{2}-y^{2}}$ and $d_{3z^{2}-r^{2}}$ orbitals along the interface that correlates specific spin and orbital states. Figure 6A displays a sketch of the simplest and most likely case of a combined AF and checkerboard-type orbital order for which the spin-up states are correlated with the $d_{x^{2}-y^{2}}$ orbitals and the down spins with the $d_{3z^{2}-r^{2}}$ orbitals (or vice versa). Figure 6B shows the accordingly calculated momentum dependence of the intensity of the magnon mode (solid line) for which the individual scattering amplitudes of the $d_{x^{2}-y^{2}}$ and $d_{3z^{2}-r^{2}}$ orbitals have been derived from a single ion model, as described in Section S4.3. Notably, this model of a combined AF and checkerboard orbital order reproduces very well the anomalous increase of the intensity of the M1 mode toward small wave vectors. Note that within this simple spin-wave model, the additional orbital order merely affects the scattering amplitudes of the spin-up and spin-down components of the AF order of the interfacial layer. The corresponding effect on the exchange interaction of the interfacial CuO_2_ layers, and on the resulting dispersion of the M1 mode, has been already accounted for in terms of the reduced value of $J_{∥}^{\mathrm{IF}}$, as discussed in the previous section. Also note that the energy and intensity dispersion of the M2 mode from the bulk-like CuO_2_ layers is hardly affected by the interfacial orbital order, due to the very weak interlayer exchange interaction and the resulting quasi-2D spin-wave properties. Accordingly, in the relevant high-energy range above 50 meV, the energy dispersion of the M1 and M2 modes as shown in Fig. 5A and B, as well as the intensity variation of the M2 mode in Fig. 5C and D, are not noticeably affected by this interfacial orbital order.

Naturally, the required disparity of the scattering amplitudes for the spin-up and spin-down components of the AF order could also be caused by a more complex orbital order or even by another type of order, such as a charge order which modulates the density of holes on the spin-up and spin-down sites. Nevertheless, we consider the orbital order described in Fig. 6A to be the most likely candidate, especially since it conforms with the more or less equal number of holes on the $d_{3z^{2}-r^{2}}$ and $d_{x^{2}-y^{2}}$ orbitals of the interfacial CuO_2_ layer that is suggested by the TEY-XAS spectra in Fig. 1C.

Irrespective of the detailed nature of the additional electronic order of the interfacial CuO_2_ layer, it turns out that all the relevant combined AF and electronic (charge or orbital) orders should give rise to a so-called altermagnetic state (54). Such altermangets are compensated, collinear magnets which exhibit a characteristic spin-splitting of the Fermi-surface with nodes at which the polarization changes its sign, such that the average spin-polarization around the Fermi-surface vanishes. Notably, this spin-splitting can be sizable irrespective of the spin-orbit-coupling strength. The combined AF and checkerboard orbital order shown in Fig. 6A corresponds to a so-called d-wave magnet for which the spin-splitting of the Fermi-surface exhibits a d-wave symmetry. Here, it is evident from symmetry considerations that the difference in energy of the $d_{3z^{2}-r^{2}}$ and $d_{x^{2}-y^{2}}$ states (in an orthorhombic or tetragonal environment) and of the respective wave functions with spin up and spin down results in an anisotropic spin polarization in momentum space.

## Summary and conclusion

In summary, we performed a high-resolution and polarization-resolved RIXS study at the Cu-L_3_ edge to investigate the magnetic and orbital orders at the YBa_2_Cu_3_O_7_/${\mathrm{Nd}}_{0.65}$(Ca,Sr)_0.35_MnO_3_ interface. We found that the RIXS spectra contain two distinct magnon modes, denoted as M1 and M2, that can be assigned to the CuO_2_ layers right at the interface (M1 mode) and those located further away from it (M2 mode), according to their characteristic resonance energies at 930.5 and 931 eV, respectively. By comparing the measured magnon dispersion with calculations based on a minimal linear spin-wave model, we confirmed that the M2 mode is well described with a bulk-like parameter of the in-plane exchange interaction $J_{∥}\approx 130$ meV. The corresponding fit of the energy dispersion of the M1 mode yields a strongly reduced value of the in-plane exchange coupling of the interfacial CuO_2_ layer of $J_{∥}^{\mathrm{IF}}\approx 70$ meV. We have outlined that such a strong suppression can be readily understood in terms of the orbital reconstruction which, according to the XAS data, yields similar amounts of holes with $d_{x^{2}-y^{2}}$ and $d_{z^{2}}$ orbital character. This results in a high density of Cu($d_{3z^{2}-r^{2}}$) −O−Cu($d_{x^{2}-y^{2}}$) bonds for which the hopping probability (and thus the exchange interaction) is strongly reduced when compared with the Cu($d_{x^{2}-y^{2}}$) −O−Cu($d_{x^{2}-y^{2}}$) bonds of bulk-like YBCO. Nevertheless, a major discrepancy with respect to the prediction of our minimal spin-wave model has been observed for the momentum dependence of the intensity of the M1 mode, which has been found to strongly increase toward small momentum transfer even though a strong suppression is predicted by the model. We have outlined that this contradiction can be readily resolved in terms of an additional electronic or orbital order that accompanies the AF order. This additional order needs to be of a specific kind such that it yields different amplitudes of the spin-up and spin-down components of the RIXS matrix elements as to overcome the destructive interference from their 180 °-phase shift toward small wave vectors that occurs for the plain AF order. As a likely candidate, that naturally accounts for the unusual momentum dependence of the M1 mode intensity, we have identified a combined AF and checkerboard order of the $d_{x^{2}-y^{2}}$ and $d_{3z^{2}-r^{2}}$ holes. This checkerboard orbital order conforms with the XAS data which suggest that the $d_{3z^{2}-r^{2}}$ and $d_{x^{2}-y^{2}}$ orbital character of the holes in the interfacial CuO_2_ layer is almost equally balanced. Nevertheless, there exist other types of orbital orders, or orders that involve the charge rather than the orbital degree of freedom, that can give rise to a strong disparity of the scattering amplitudes of the up and down spins of the AF state and thus can explain the anomalous intensity evolution of the M1 mode. However, it turns out that all of the relevant cases give rise to a combined AF and charge or orbital order which has the properties of a so-called altermagnetic state (54).

Such altermagnetic orders have recently obtained a great deal of attention since they exhibit a characteristic spin-splitting of the Fermi-surface with nodes at which the sign of the polarization changes. For the specifically discussed AF and checkerboard orbital order shown in Fig. 6A, the spin-splitting of the Fermi-surface varies according to a d-wave symmetry pattern such that it can be classified as a quasi-2D d-wave magnet. The electronic and magnetic properties of such altermagnets are a fairly new research field that is rapidly growing since they offer unique opportunities for new kinds of spintronic and magneto-calorimetric applications (54–56). For example, the directional dependence of their spin-polarization effects in transport and tunneling has been predicted to enable new device functionalities. It has also been pointed out that the interplay between an altermagnet and a superconductor can give rise to unconventional proximity effects or even to a new kind of SC pairing mechanism (57, 58). Our observation that the CuO_2_ layers at a cuprate/manganite interface seem to host a quasi-2D altermagnetic state, that is in direct proximity to the high-$T_{c}$ SC order in the neighboring CuO_2_ layer, is therefore of great current interest. In the first place, it calls for the fabrication of suitable devices that enable transport studies of the directional anisotropy of the SC currents and/or of the spin polarization of the normal currents, in order to confirm the altermagnetic properties of this cuprate/manganite interface.

Hopefully, our results will also simulate further studies of the origin of the orbital ordering of the interfacial Cu ions. At present, we can only speculate that it might be driven by a buckling of the interfacial Ba–O layer which helps to accommodate the lattice mismatch between the cuprate and manganite layers. Alternatively, it may be triggered by a corresponding charge/orbital order on the manganite side of the interface. The latter scenario could be further explored with complementary RIXS studies at the Mn-L_3_ edge. Of great interest would also be corresponding studies of YBCO/manganite heterostructures for which the manganite layers have a higher doping level of x=0.5 and host a well-developed combined CE-type AF and charge/orbital order. Notably, for such heterostructures, the XAS experiments have indicated weaker charge transfer and orbital reconstruction effects (24). Moreover, a previous RIXS study has revealed a charge order with a period of about 10 unit cells that appears to have a $d_{z^{2}}$ orbital character (24) which clearly distinguishes it from the charge density wave of bulk YBCO that involves primarily the $d_{x^{2}-y^{2}}$ states.

Last but not least, our results have highlighted the potential of the high-resolution RIXS technique to selectively probe the magnetic properties at the interfaces of other kinds of magnetic multilayers. Potential candidates are heterostructures from complex oxides that are known to exhibit various kinds of magnetic orders (34, 59, 60), or from other materials with versatile electronic and magnetic properties that are strongly modified in the vicinity of their interfaces, such as the transition metal chalcogenides or related van der Waals materials (61, 62).

## Experimental methods

### Sample preparation and characterization

The sample was deposited by Pulsed Laser Deposition. A detailed description of the growth conditions and its characterization is presented in Ref. (24).

### X-ray absorption spectroscopy

The XAS, XLD, and XMCD (Figs. 1, S2, and S3) were measured at the XMCD endstation of the ID 32 beamline at ESRF, Grenoble in France (63), in total fluorescence yield (FY) mode and TEY mode, at the Cu-L_3_-edge at 20 K. The TFY detector is a photodiode shielded with an Al foil, in order to avoid electrons reaching the detector. The incidence angle was fixed to $30^{\circ }$ with respect to the incoming beam, which was horizontally or vertically polarized. The resolution of the acquisition was 110 meV. Multiple scans were averaged to produce the final plots in Figs. 1, S2, and S3. The horizontal spot size of the beam was ≈30  μm.

### RIXS at I21 beamline in DLS, Oxford

RIXS experiments at the Cu-L_3_ edge with a very high-energy resolution of ∼42 meV have been performed using the RIXS spectrometer at the I21 beamline of the DLS, in Oxford, UK (64). The measurements were performed in grazing exit geometry at 20 K. For every scan, the position of the elastic line, the energy to pixel ratio, and the resolution have been determined from a reference measurement on a carbon tape (which acts as a nonresonant scatterer). The obtained energy calibration was 6.7 meV/pixel. The size of the beam-spot was about 2 μm in the vertical direction and 40 μm in the horizontal direction.

### RIXS–polarimetry at ID32 beamline in ESRF, Grenoble

The RIXS–polarimetry experiments at the Cu-L_3_ edge have been performed at the ID32 beamline of the ESRF, in Grenoble (51), France. Here, the polarization of the scattered beam has been analyzed to determine the magnetic spin-flip scattering. The polarization of the scattered X-ray photons was determined by inserting in the path of the analyzed beam a mirror with a different reflectivity for *σ* and *π* polarization ($R_{\sigma }=0.141$, $R_{\pi }=0.086$) and comparing the scattered intensity to that without the mirror. Further details are given in Section S3.1 (40). For each scan, the position and resolution of the zero-loss position has been tracked using a nonresonant scatterer. The energy calibration for with and without the mirror was 21.471 and 21.306 meV/pixel, respectively. The beam-spot size was 2 μm in the vertical direction and 40 μm in the horizontal direction.

## Supplementary Material

pgae100_Supplementary_Data

## Data Availability

The XAS data are available at https://doi.esrf.fr/10.15151/ESRF-ES-511177236. The RIXS data from ESRF are available at https://doi.esrf.fr/10.15151/ESRF-DC-1314300736 and https://doi.esrf.fr/10.15151/ESRF-ES-518219483. The RIXS data from DLS are available at https://doi.org/10.5281/zenodo.8283049.
