# Supplementary material for: Composite antiferromagnetic and orbital order with altermagnetic properties at a cuprate/manganite interface
Source: PNAS Nexus. 2024 Mar 4;3(4):pgae100. doi: 10.1093/pnasnexus/pgae100 (PMC11081879; doi:10.1093/pnasnexus/pgae100)
Supplement: pgae100_Supplementary_Data [file pgae100_supplementary_data.pdf]

Supporting Information for

Composite antiferromagnetic and orbital order with altermagnetic properties at a cuprate/manganite interface

Subhrangsu Sarkar, Roxana Capu, Yurii G. Pashkevich, Jonas Knobel, Marli Dos Reis Cantarino, Abhishek Nag, Kurt Kummer, Davide Betto, Roberto Sant, Christopher W. Nicholson, Jarji Khmaladze, Ke-Jin Zhou, Nicholas B. Brookes, Claude Monney, Christian Bernhard

Paste corresponding author name here
Email: subhrangsu.sarkar@unifr.ch, roxana.capu@e-uvt.ro, christian.bernhard@unifr.ch

This PDF file includes:

Supporting text
Figures S1 to S8
SI References

Supporting Information Text

S1. X-ray absorption Spectroscopy

S1.1. Linear dichroism and multi-peak fitting:

For each polarization of the incident X-ray beam, the experimental spectra are first averaged and a linear background is subtracted such that the intensity well below the Cu-L_3 edge vanishes. Subsequently, the intensity is normalized to yield a unity value for the edge jump above the Cu-L_3 edge. The spectrum for linear σ polarization of the incident X-ray beam represents the response along the ab-plane of YBCO, μ_{ab}. The corresponding c-axis response, μ_c, has been derived from the measured spectrum in linear π-polarization, μ_π, and the incidence angle of the x-ray beam of 30° according to the following equation(1):

$$\mu_c = 1/\left(\cos^2(30°)\mu_\pi - \tan^2(30°)\mu_{ab}\right)$$

The corresponding polarization-averaged absorption, μ_{avg}, amounts to:

$$\mu_{avg} = \max\frac{(2\mu_{ab} + \mu_c)}{3}$$

In Fig. 1 in the main manuscript, the solid symbols represent the experimental data and solid lines show the best fits with Lorentzian functions. The colored shading indicates when $\mu_{ab} > \mu_c$ ($\mu_{ab} < \mu_c$) and the difference ($\mu_{ab} - \mu_c$) is shaded in blue (red) indicating a positive (or negative) value of the x-ray linear dichroism. During the fits, we fixed the position of the peaks in the FY and TEY spectra with a maximal deviation of 0.1eV and fitted both of them concomitantly.

S1.2. XMCD spectra:

The X-ray magnetic circular dichroism (XMCD) signal (also measured at an incidence angle of 30°) is proportional to the difference between the normalized spectra measured with right circular polarization, μ_+, and left circular polarization, μ_-, (measured at same temperature and applied magnetic field) according to the following equation:

$$XMCD = \frac{(\mu_+ - \mu_-)}{\frac{1}{2}(\mu_+ + \mu_-)} \times 100$$

Here, $\frac{1}{2}(\mu_+ + \mu_-)$ corresponds to the XANES spectrum that is shown in the Fig S2 and S3 for the measurements in TEY (Cu and Mn L3-edge) and FY (Cu-L3 edge) modes.

S1.3. Experimental observation of hysteretic Cu-moment at the interface:

The Cu-XMCD data in Fig. S2 reveal a weak FM Cu moment that originates from the interfacial Cu ions. This assignment is based on the distinct resonance energies of the bulk-like and the interfacial Cu ions at 931 eV and 930.5 eV, respectively, which are evident from the XAS spectra in FY and TEY mode shown in Fig. S2A. Fig. S2B reveals that the Cu-XMCD signal is indeed peaked around 930.5 eV and thus at the resonance of

the interfacial Cu ions. Figs. S3 confirms that in FY mode this Cu-XCMD signal is also peaked at 930.5 eV and also considerably weaker than in TEY mode.

The FM Cu moment can be understood in terms of a canting of the planar AF order of the interfacial CuO_2 layer that is induced by an AF exchange interaction with the FM Mn moments on the manganite side of the interface. The energy minimization in Heisenberg approximation suggests that the induced Cu moment is perpendicular to the Néel vector and lying within the CuO_2 plane, as sketched in Fig. S3D.

The AF nature of the exchange interaction and the subsequent antiparallel alignment of the Cu moment with respect to the Mn moment is evident from the opposite signs of the Cu- and Mn-XMCD signals in a small magnetic field of 0.5 Tesla shown in Figs S2B and S2F, respectively. The magnitude of J_{MC} can be estimated from the characteristic magnetic field dependence of the Cu-XMCD signal in Figs S2B and S2C which exhibits a sign reversal at a critical field of $H_c \sim 3-4$ Tesla above which it becomes positive as the Zeeman energy overcomes the AF exchange coupling, J_{MC} , and thus causes a reorientation of the Cu moment. According to our model calculations (see Eq.S4.5 below) this crossing point thus yields an estimate of $J_{MC} \sim \mu_B \times H_c \sim 0.5$ meV.

Such a small value of J_{MC} is also suggested by the analogy with the exchange coupling between Cu spins on the planar and the chain sites of the undoped and antiferromagnetic $YBa_2Cu_3O_6$, which is theoretically predicted around 1 meV(2).

Notably, a corresponding field-induced reorientation of the Cu moment was observed in $YBCO/La_{2/3}Ca_{1/3}MnO_3$ heterostructures in Ref.(3), where it was shown that the strength of the interfacial exchange coupling J_{MC} can vary substantially for samples with different electronic and magnetic properties of the LCMO layers. Moreover, it was previously shown with XRMR that the Cu moments that give rise to the FM order are located on the cuprate side of the interface and thus do not arise from interdiffusion of some Cu ions across the interface during the PLD growth(4). A more detailed understanding of how J_{MC} and the induced FM Cu moment vary with the electronic and magnetic properties of the manganite layers, that can be strongly modified via the hole doping or the tolerance factor, is still lacking.

For the present YBCO/NCSMO superlattice, the hole doping and the tolerance factor of the NCSMO layers are chosen such that they are very close to a phase transition between a FM metallic state (at lower hole doping and high magnetic field) and a predominantly AF and charge/orbital ordered insulating state (toward higher hole doping and low magnetic field). As was shown in Fig. 2 of Ref.(5), and is confirmed by the Mn-XMCD spectra in TEY mode in Fig. S2(f), the NCSMO layers of the present superlattice exhibit a sizeable FM Mn moment with very low field of saturation (around 0.5 T). This behavior is typical for soft ferromagnets and it excludes a possible origin of the manganite FM moment from strong canting of the AF Mn moments. Whether this FM Mn moment arises from a phase separation into AF and FM regions or from a strong alteration of the

magnetic properties of the MnO_2 layers in the vicinity of the cuprate/manganite interface is not established yet.

S2. Normalization, self-absorption correction, and fitting of the RIXS spectra

S2.1. Normalization and self-absorption correction of the raw data

The raw counts obtained from the detector were first normalized to the input drain current (which is a measure of incident x-ray flux). The data after this normalization are presented in Fig S4.

Subsequently, a self-absorption correction has been performed that accounts for the change of the interaction volume between the x-ray and the sample (film) that occurs as the Q value respectively the incidence angle of the x-rays is varied. For this self-absorption correction, which plays an important role in the intensities of the inelastic features, we have followed the procedure described in ref.(6, 7). As an example, Fig S5.a shows a comparison of an initial and a self-absorption corrected RIXS spectrum along $[h, 0]$ at 0.33a*.

Finally, to correct for the different footprints of the incident x-ray beam at different incidence angles, the self-absorption corrected RIXS spectra have been normalized to the area of the *dd*-excitations as shown in Fig S5.b. Note that the area of the *dd*-excitation is proportional to the total scattering cross-section of the sample with the incident x-ray which does not depend on the incidence angle.

S2.2. Fitting of the normalized data.

The normalized and self-absorption corrected spectra (as described in section S2.1) have been subsequently fitted with a multi-peak model. As shown in Fig S6, the spectra have been reproduced with (i) 3 peaks to account for the strong *dd*-excitations, consistent with e.g. ref (8), (ii) an elastic peak at zero energy-loss that has a width similar to that of the experimental resolution of 42 meV, (iii) two peaks to account for the high energy phonon-modes (the so-called buckling and stretching mode(9)), (iv) two peaks to account for the magnon modes and (v) one weak peak due to a bimagnon. In addition, we have included a very broad background that accounts for an extension of the high-energy charge-transfer peak. Finally, we have included an additional weak peak around an energy loss of –0.7 eV which arises from the orbital reconstruction effect of the interfacial CuO_2 layer that shifts the $d_{x^2-y^2}$.

The magnon bands were fitted with the following equations:

For E< -0.001,

$$\tilde{S} = 2E_0\tilde{\chi}\tilde{\Gamma}\frac{E}{\left[1-e^{\frac{E}{k_BT}}\right]\left[(E^2-E_0^2)^2+\left(E\tilde{\Gamma}\right)^2\right]} \qquad \text{(Eq. S2.1)}$$

Otherwise,

$$\tilde{S} = 2E_0\tilde{\chi}\tilde{\Gamma}\frac{E}{\left[1-e^{\frac{E-0.001}{k_BT}}\right]\left[(E^2-E_0^2)^2+\left(E\tilde{\Gamma}\right)^2\right]} \qquad \text{(Eq. S2.2)}$$

Here $\tilde{S}$ is the spectral response of the magnons; E_0 the center energy, $\tilde{\chi}$ *the* amplitude and $\tilde{\Gamma}$ the width of the magnon band, and E the energy loss. A more detailed motivation and description of these fit functions can be found in reference (10). The elastic peak and phonons were fitted with the following functions:

$$y = y0 + A_{el}e^{-\frac{(E-E_{el})^2}{W_{el}^2}} \qquad \text{(Eq.}S2.3)$$

$$y = y0 + A_{ph}e^{-\frac{\left(E-E_{ph}\right)^2}{W_{ph}^2}} \qquad \text{(Eq.}S2.4)$$

To account for their skewed shape, the crystal field excitations were fitted with bi-Gaussian functions:

for $x < x_c$

$$y = y0 + He^{-\frac{(x-x_c)^2}{2w_1^2}} \qquad \text{(Eq.}S2.5)$$

else

$$y = y0 + He^{-\frac{(x-x_c)^2}{2w_2^2}} \qquad \text{(Eq.}S2.6)$$

To describe the bimagnons and a weak peak around -0.7eV, that arises from a low-energy crystal field excitation that involves the $d_{3z^2-r^2}$ levels of the interfacial Cu ions that are much closer to the Fermi-level than in the bulk-like Cu ions, we used the following Gaussian function:

$$y = y0 + \left(\frac{A}{w\sqrt{\left(\frac{\pi}{2}\right)}}\right)e^{-2\left(\frac{x-xc}{w}\right)^2} \qquad \text{(Eq.}S2.7)$$

S2.3. Calculation of error bars in magnon-parameters

The error bars in Fig. 4 of the main manuscript have been estimated as follows:

1. Error in position and width: The maximum error in position and width has been estimated to be the resolution of the spectrometer during the experiment: 42 meV.
2. Error in the Area of the magnon peaks: The Area of the magnon peaks can be estimated as:

$$Area_{M1,M2} = (constant) \times height\ of\ the\ peak\ (ht) \times withdth\ of\ the\ peak(w)$$

Consequently, the error in area is estimated as:

$$\delta\left(Area_{M1,M2}\right) = \left|Area_{M1,M2}\right| \times \sqrt{\left(\frac{\delta ht}{ht}\right)^2 + \left(\frac{\delta w}{w}\right)^2}$$

Here, $\delta ht = RMSD = \sqrt{\frac{1}{N}\sum_N (Fit - Exp.data)^2}$ and δw=42 meV

It is worth mentioning that the error bars estimated here provide the maximum value of error instead of an expectation of error. Following the supplementary info in ref.(11), one can assume that the error in position and width after a successful fit is approximately 10 times smaller than the instrumental resolution. However, since the fitting function for magnons in our analysis is much more complex, we prefer to use the maximum limit of the errors in estimating the error bars.

S3. Polarization analysis of the RIXS intensity and error bar calculation

S3.1. Polarization dependence of scattered x-rays:

As sketched in Fig. 2A, in a polarimetry experiment one measures (with σ- and π-polarized incident photons) the RIXS intensities after reflection from of a multilayer mirror, I_{pol}, and references it to the scattered intensity of the direct beam without the multilayer mirror, I_{dir}. With the predetermined values of the polarization-dependent reflectivity of the multilayer-mirror (of $R_\pi = 0.086 \pm 0.002, and\ R_\sigma = 0.141 \pm 0.002$ at Cu L3 edge), one can thus deduce the $I_\pi -$ and $I_\sigma -$components of the RIXS signal according to the following relationship:

$$I_\pi = \frac{R_\sigma\, I_{dir} - I_{pol}}{R_\sigma - R_\pi}; \qquad I_\sigma = \frac{I_{pol} - R_\pi\, I_{dir}}{R_\sigma - R_\pi} \qquad \text{(Eq. S3.1)}$$

S3.2. Error bar calculation:

The RIXS intensity has been obtained by normalizing the scattered intensity with the input drain current (proportional to the incident x-ray flux) measured from a reference mirror M_j that is placed on the input side of the spectrometer:

$$I_{RIXS} = \sum_j \frac{I_j}{M_j} \qquad \text{(Eq. S3.2)}$$

For simplicity it is assumed that M_j is almost constant and thus can be approximated by the averaged mirror current M, such that the error of the RIXS intensity can be calculated as:

$$\Delta I_{RIXS} = \frac{1}{M}\sqrt{\sum I_j} \qquad \text{(Eq. S3.3)}$$

We typically made sure that the counts per channel in the single photon counter (SPC) is greater than 20 photons so the distribution can be approximated with a gaussian function for which $\Delta I_{dir,pol} \approx \sqrt{I_{dir,pol}}$. Accordingly, the error bars for the scattered intensities can be calculated using the following standard error propagation:

$$(\Delta I_\pi)^2 \approx \left(\frac{\partial I_\pi}{\partial I_{dir}}\right)^2 (\Delta I_{dir})^2 + \left(\frac{\partial I_\pi}{\partial I_{pol}}\right)^2 (\Delta I_{pol})^2 + \left(\frac{\partial I_\pi}{\partial R_\pi}\right)^2 (\Delta R_\pi)^2 + \left(\frac{\partial I_\pi}{\partial R_\sigma}\right)^2 (\Delta R_\sigma)^2 +$$

$$= \left(\frac{R_\sigma}{R_\sigma - R_\pi}\right)^2 (\Delta I_{dir})^2 + \left(\frac{-1}{R_\sigma - R_\pi}\right)^2 (\Delta I_{pol})^2 + \left(\frac{R_\sigma I_{dir} - I_{pol}}{(R_\sigma - R_\pi)^2}\right)^2 (\Delta R_\pi)^2$$

$$+ \left(\frac{I_{dir}}{R_\sigma - R_\pi} - \frac{R_\sigma I_{dir} - I_{pol}}{(R_\sigma - R_\pi)^2}\right)^2 (\Delta R_\sigma)^2$$

$$(\Delta I_\sigma)^2 \approx \left(\frac{-R_\pi}{R_\sigma - R_\pi}\right)^2 (\Delta I_{dir})^2 + \left(\frac{1}{R_\sigma - R_\pi}\right)^2 (\Delta I_{pol})^2 +$$

$$\left(\frac{I_{pol} - R_\pi I_{dir}}{(R_\sigma - R_\pi)^2} - \frac{I_{dir}}{R_\sigma - R_\pi}\right)^2 (\Delta R_\pi)^2 + \left(\frac{R_\sigma I_{dir} - I_{pol}}{(R_\sigma - R_\pi)^2}\right)^2 (\Delta R_\sigma)^2 \qquad \text{(Eq. S3.4)}$$

S4. Theoretical estimate of magnon dispersion and RIXS intensity

S4.1. Description of the linear spin wave model and the AF ground state.

We apply a minimal linear spin wave model with a special contribution from the interfacial CuO_2 layer to obtain a description of the two magnon modes (M1 and M2) that are observed in the RIXS spectra. Specifically, we adopt a 2D Heisenberg nearest neighbor model for the CuO_2 double layers, where only the interfacial CuO_2 layer is affected by a weak, antiferromagnetic (AF) exchange interaction, J_{MC}, with the Mn moments on the other side of the interface (the latter are assumed to exhibit a soft ferromagnetic order). In addition, we consider that the charge transfer and the related orbital reconstruction at the interface leads to a strong decrease in the population of the $d_{x^2-y^2}$ orbitals and a corresponding increase of the $d_{3z^2-r^2}$ orbitals for the interfacial CuO_2 layer *(12)*. Accordingly, we allow for a reduced in-plane AF exchange $J_{\parallel}^{IF}$ of the interfacial CuO_2 plane**.** For the other CuO_2 plane of this interfacial bilayer-unit, our minimal model assumes that $J_{\parallel}$ recovers already a bulk-like value. This is to reduce the number of fit parameters and motivated by our finding that the RIXS spectra show no sign of a third magnon peak. For the interplanar antiferromagnetic exchange $J_\perp$ between the adjacent CuO_2 layers of these bilayer units, we also adopt a bulk-like value.

A sketch of our model is depicted in Fig. 2B of the main text. Here we assume a twinned order of the orthorhombic structure of fully oxygenated and superconducting YBCO described by the space group *Pmmm (13)* with the lattice constants *a=3.82* Å, *b*=3.88Å, *c*=11.68Å. The long range magnetic order is described by the propagation vector ***k*** = (1/2,1/2,0) *(14)* such that the primitive magnetic unit cell with the lattice constants **A** = **a+b**; **B**= **a - b**; **C**=**c** contains four magnetic copper ions with coordinates Cu1 =(0,0,z), Cu2 = (1/2, ½, -z), Cu3 (0,0,-z); and Cu4 = (1/2,1/2, z) with z = 0.355(8). The Cu1 and Cu4 ions belong to the interfacial CuO_2 plane. It is supposed that the Neel antiferromagnetic

vector of the copper bilayer is directed along the $\boldsymbol{a}$ – axis and the ferromagnetic moment of the manganite interfacial plane is directed along the $\boldsymbol{b}$ -axis. The external magnetic field is applied along the direction of the ferromagnetic manganite moment.

The respective Hamiltonian of our model in exchange approximation has the form:

$$\hat{H}^{(ex)} = \sum_{n,m}\left[J_{\parallel}(\boldsymbol{s}_{2n}\boldsymbol{s}_{3m}) + J_{\parallel}^{IF}(\boldsymbol{s}_{1n}\boldsymbol{s}_{4m})\right] + \sum_{n} J_{\perp}(\boldsymbol{s}_{1n}\boldsymbol{s}_{3n} + \boldsymbol{s}_{4n}\boldsymbol{s}_{2n}) + \\ + \sum_{n} J_{MC}\boldsymbol{n}_{Mn}(\boldsymbol{s}_{1n} + \boldsymbol{s}_{4n}) - \sum_{n} g\mu_B \boldsymbol{H}(\boldsymbol{s}_{1n} + \boldsymbol{s}_{2n} + \boldsymbol{s}_{3n} + \boldsymbol{s}_{4n}) \qquad \text{(Eq. S4.1)}$$

Here $J_{\parallel}$, $J_{\perp}$,$J_{\parallel}^{IF}$,J_{MC} > 0. In the following calculation, we assume that the vector $\boldsymbol{n}_{\mathrm{Mn}}$ = (0,1,0) is directed along the manganite's ferromagnetic moment, such that the value of J_{MC} includes the spin of the Mn ions at the interface.

Further, to make the symmetry properties of the calculated results more transparent, we introduce the following linear combinations of the Fourier components of the sublattice spins:

$$\begin{aligned} \boldsymbol{F}(\boldsymbol{k}) &= \boldsymbol{s}_1(\boldsymbol{k}) + \boldsymbol{s}_2(\boldsymbol{k}) + \boldsymbol{s}_3(\boldsymbol{k}) + \boldsymbol{s}_4(\boldsymbol{k})\,; \\ \boldsymbol{L}_1(\boldsymbol{k}) &= \boldsymbol{s}_1(\boldsymbol{k}) + \boldsymbol{s}_2(\boldsymbol{k}) - \boldsymbol{s}_3(\boldsymbol{k}) - \boldsymbol{s}_4(\boldsymbol{k})\,; \\ \boldsymbol{L}_2(\boldsymbol{k}) &= \boldsymbol{s}_1(\boldsymbol{k}) - \boldsymbol{s}_2(\boldsymbol{k}) + \boldsymbol{s}_3(\boldsymbol{k}) - \boldsymbol{s}_4(\boldsymbol{k})\,; \\ \boldsymbol{L}_3(\boldsymbol{k}) &= \boldsymbol{s}_1(\boldsymbol{k}) - \boldsymbol{s}_2(\boldsymbol{k}) - \boldsymbol{s}_3(\boldsymbol{k}) + \boldsymbol{s}_4(\boldsymbol{k}). \end{aligned} \qquad \text{(Eq. S4.2)}$$

Rewriting Hamiltonian (Eq. S5.1) in terms of linear combinations (Eq. S5.2), we get:

$$\begin{aligned} \hat{H}^{(ex)} = \Sigma\{&A(\boldsymbol{k})[\boldsymbol{F}(\boldsymbol{k})\boldsymbol{F}(-\boldsymbol{k}) - \boldsymbol{L}_1(\boldsymbol{k})\boldsymbol{L}_1(-\boldsymbol{k})] + B(\boldsymbol{k})[\boldsymbol{L}_2(\boldsymbol{k})\boldsymbol{L}_2(-\boldsymbol{k}) \\ &- \boldsymbol{L}_3(\boldsymbol{k})\boldsymbol{L}_3(-\boldsymbol{k})] + C(\boldsymbol{k})[\boldsymbol{F}(\boldsymbol{k})\boldsymbol{L}_3(-\boldsymbol{k}) - \boldsymbol{L}_1(\boldsymbol{k})\boldsymbol{L}_2(-\boldsymbol{k})]\} \\ &+ \frac{1}{2}\sqrt{N}J_{MC}[F_y(0) + L_{3y}(0)] - \sqrt{N}g\mu_B H F_y(0); \end{aligned} \qquad \text{(Eq. S4.3)}$$

Here N is the number of the unit cells in the crystals and the *A, B, and C* coefficients are defined as:

$$\begin{aligned} A(\boldsymbol{k}) &= \frac{1}{8}\left[J_{\perp}(\boldsymbol{k}) + \frac{1}{2}J_{\parallel}(\boldsymbol{k}) + \frac{1}{2}J_{\parallel}^{IF}(\boldsymbol{k})\right]; \\ B(\boldsymbol{k}) &= \frac{1}{8}\left[J_{\perp}(\boldsymbol{k}) - \frac{1}{2}J_{\parallel}(\boldsymbol{k}) - \frac{1}{2}J_{\parallel}^{IF}(\boldsymbol{k})\right]; \\ C(\boldsymbol{k}) &= \frac{1}{8}[J_{\parallel}(\boldsymbol{k}) - J_{\parallel}^{IF}(\boldsymbol{k})]; \end{aligned} \qquad \text{(Eq. S4.4)}$$

with

$J_{\parallel}(\boldsymbol{k}) = 2J_{\parallel}[cos\,\boldsymbol{k}\,\boldsymbol{a} + cos\,\boldsymbol{k}\,\boldsymbol{b}]$; $J_{\parallel}^{IF}(\boldsymbol{k}) = 2J_{\parallel}^{IF}[cos\,\boldsymbol{k}\,\boldsymbol{a} + cos\,\boldsymbol{k}\,\boldsymbol{b}]$;
$J_{\perp}(\boldsymbol{k}) = J_{\perp}\,cos\,\boldsymbol{k}_{\perp}\,\boldsymbol{d}$;

In our model the Neel vector (magnetic order parameter) of the bulk-like YBCO is directed along the $\boldsymbol{a}$ – axis. Thus, the magnetic ground state is described by the nonzero Neel vector $\bar{L}_{1x} = 4S$ where S is the spin of the copper ions. The magnetic excitations (with four branches of spin waves) of our double layer model for bulk YBCO are described by the set of operators S4.2. In particular, the spin operators L_{1y} , F_z and L_{1z} , F_y determine the deviation of the primary order parameter from its equilibrium state. In the absence of anisotropy, the corresponding spin waves are gapless Goldstone modes (AM - acoustic modes). For small wave vectors $\boldsymbol{k}$, the AM1 (L_{1y} , F_z) and AM2 (L_{1z} , F_y) modes are mainly in-plane (L_{1y}) and out-of-plane (L_{1z}) high-amplitude fluctuations of the Neel vector, respectively. In the exchange approximation at $\boldsymbol{k}$ = 0 their amplitudes diverge. Their fluctuations become less pronounced with increasing $\boldsymbol{k}$.

Another type of spin operators is due to the gap magnetic excitations that break a given type of exchange magnetic order, which therefore requires some exchange energy (OM - optical modes). In our double layer model, the respective gap at $\boldsymbol{k}$=0 is proportional to $\varepsilon_{OM}(0) \propto \sqrt{J_{\perp}J_{\parallel}}$ *(14)*. The sets of operators L_{3y}, L_{2z} and L_{2y}, L_{3z} describe the OM1 and OM2 optic modes respectively. In the exchange approximation for bulk YBCO ($J_{MC} = 0; J_{\parallel}^{IF} = J_{\parallel}; H = 0$,), the acoustic and optic spin waves do not mix with each other at any $\boldsymbol{k}$ ≠ 0 in the magnetic Brillouin zone.

The reduced intraplanar exchange $J_{\parallel}^{IF} < J_{\parallel}$, as well the effects of the exchange with the Mn moments, J_{MC}, and with the external magnetic field (applied along the b-axis) all tend to decrease the initial magnetic symmetry and give rise to a mixing of the AM1 and OM2 and the AM2 and OM1 modes. However, the main mixing effect arises from the strong reduction of the intraplanar AFM exchange of the interfacial CuO_2 layer $J_{\parallel} - J_{\parallel}^{IF}$ that turns out to have by far the largest magnitude of about 60 meV. The respective terms in the Hamiltonian S4.3 are proportional to the coefficient $C(\boldsymbol{k})$.

The magnetic ground state changes according to the new symmetry. Static nonzero components $\bar{F}_y$, $\bar{L}_{1z}$,$\bar{L}_{3y}$, $\bar{L}_{2z}$, $\bar{L}_{2x}$ arise now due to the symmetry breaking by the interfacial manganite layer. Here the y- and z-components describe complex in-plane and out-of-plane tilts of the initial magnetic structure. In the following, we neglect the out-of-plane tilts as they arise as secondary effects caused by the Dzyaloshinskii-Moriya interaction, $D_1F_yL_{1z} + D_2L_{3y}L_{2z}$, which we do not consider in our Heisenberg model. The remaining components $\bar{F}_y$ *and* $\bar{L}_{3y}$ determine the angles of the tilts from the x- towards the y-axis: θ_1 – for the magnetic sublattices 1 and 4 of the interfacial CuO_2 plane and θ_2 – for the magnetic sublattices 2 and 3 of the second CuO_2 of that bilayer unit. The minimization of the energy thus yields the following relationships:

$$\frac{1}{2}(\bar{F}_y + \bar{L}_{3y}) = \bar{S}_{1y} + \bar{S}_{4y} = 2S\,sin\,\theta_1 = \frac{-J_{MC} + g\mu_B H}{4(J_{\parallel}^{IF} + \frac{J_{\perp}}{8})};$$

$$\frac{1}{2}(\bar{F}_y - \bar{L}_{3y}) = \bar{S}_{2y} + \bar{S}_{3y} = 2S\,sin\,\theta_2 = \frac{g\mu_B H - J_{\perp} S\,sin\,\theta_1}{4J_{\parallel}}; \quad \text{(Eq. S.4.5)}$$

In zero magnetic field, H, the induced magnetic moment of the interfacial Cu ions has a magnitude, $m_{in} = \frac{\mu_B\,sin\,\theta_1}{Cu}$ and is antiparallel to the direction of the ferromagnetic manganite moment. As an external magnetic field is applied, the interfacial Cu moment eventually changes sign at the critical external field, $g\mu_B H_c = J_{MC}$. Our experimental XMCD data in Fig S2 in the main text yield a rough estimate of $H_c \approx 4$ T and thus $J_{MC} \approx 0.5$meV. According to Eq. S4.5, the reduction of $J_{\parallel}^{IF}$ enhances the magnetization of the interfacial CuO_2 layer.

The expected tilting angle θ_2 of the Cu2 and Cu3 sublattices in the second copper plane is expected to be opposite and much less than θ_1, since the action of the manganite ferromagnetic moment transfers from the interfacial copper plane to the second copper plane through the AFM interplane exchange $J_{\perp} << J_{\parallel}$. The respective quantitative result is shown in Eq. S4.5. In the following calculations we neglect this weak tilting in the magnetic sublattices 2 and 3 of the second copper plane.

The magnetic moments on the copper ions induced by the external magnetic field in the bulk layers ($J_{MC} = 0; J_{\parallel}^{IF} = J_{\parallel}$) can be defined by the relation:

$$m_b(Cu) = \mu_B\,sin\,\theta = \frac{g\mu_B H}{4(J_{\parallel} + \frac{J_{\perp}}{8})}\mu_B$$

Notice that this result is valid for any direction of the field since it was obtained in the exchange approximation.

S4.2. Spin wave calculations

We use a Holstein - Primakoff approach with a transformation of the sublattice spin operators to the bosonic creation and destruction operators $a_{\alpha}^{+}(\boldsymbol{k}),\ a_{\alpha}(\boldsymbol{k})$ where α =1,2,3,4 are the sublattices numbers. As a first step, we express the sublattice spin operators $s_{i\alpha}(\boldsymbol{k})$ in the crystal coordinate frame with the spin operators $s'_{i\alpha}(\boldsymbol{k})$ in a local coordinate frame for which the z'- axis is directed along the equilibrium value of the spin $\bar{s}_{\alpha}$. We use the relations $s_{i\alpha}(\boldsymbol{k}) = p_{ij}^{(\alpha)} s'_{j\alpha}(\boldsymbol{k})$, where the matrices $\hat{p}^{(\alpha)}$ have the following form:

$$\hat{p}^{(1)} = \begin{pmatrix} sin\,\theta & 0 & cos\,\theta \\ cos\,\theta & 0 & -sin\,\theta \\ 0 & 1 & 0 \end{pmatrix}; \quad \hat{p}^{(4)} = \begin{pmatrix} -sin\,\theta & 0 & -cos\,\theta \\ cos\,\theta & 0 & -sin\,\theta \\ 0 & -1 & 0 \end{pmatrix}; \quad \text{(Eq. S4.6)}$$

$$\hat{p}^{(2)} = \begin{pmatrix} 0 & 0 & 1 \\ 1 & 0 & 0 \\ 0 & 1 & 0 \end{pmatrix}; \quad \hat{p}^{(3)} = \begin{pmatrix} 0 & 0 & -1 \\ 1 & 0 & 0 \\ 0 & -1 & 0 \end{pmatrix};$$

Here $\theta = \theta_1$ and $\theta_2 = 0$. The local axial y' - axes are always directed along the z-axis of the crystal coordinate frame. With the help of Eq. S4.6 one can construct the following relations between the set of operators L in the form (Eq. S4.2) with operators L' in the same form but with spin operators $s'_{i\alpha}(\boldsymbol{k})$ from the local coordinate frame:

$$F_x(\boldsymbol{k}) = \frac{1}{2}\{sin\,\theta\,(L'_{1x}(\boldsymbol{k}) + L'_{2x}(\boldsymbol{k})) + L'_{1z}(\boldsymbol{k})(cos\,\theta + 1) + L'_{2z}(\boldsymbol{k})(cos\,\theta - 1)\};$$
$$F_y(\boldsymbol{k}) = \frac{1}{2}\{(cos\,\theta + 1)F'_x(\boldsymbol{k}) + (cos\,\theta - 1)L'_{3x}(\boldsymbol{k}) - sin\,\theta\,(F'_z(\boldsymbol{k}) + L'_{3z}(\boldsymbol{k}))\};$$
$$L_{1x}(\boldsymbol{k}) = \frac{1}{2}\{sin\,\theta\,(F'_x(\boldsymbol{k}) + L'_{3x}(\boldsymbol{k})) + F'_z(\boldsymbol{k})(cos\,\theta + 1) + L'_{3z}(\boldsymbol{k})(cos\,\theta - 1)\};$$
$$L_{1y}(\boldsymbol{k}) = \frac{1}{2}\{(cos\,\theta + 1)L'_{1x}(\boldsymbol{k}) + (cos\,\theta - 1)L'_{2x}(\boldsymbol{k}) - sin\,\theta\,(L'_{1z}(\boldsymbol{k}) + L'_{2z}(\boldsymbol{k}))\};$$
$$L_{2x}(\boldsymbol{k}) = \frac{1}{2}\{sin\,\theta\,(F'_x(\boldsymbol{k}) + L'_{3x}(\boldsymbol{k})) + F'_z(\boldsymbol{k})(cos\,\theta - 1) + L'_{3z}(\boldsymbol{k})(cos\,\theta + 1)\}; \qquad \text{(Eq S4.7)}$$
$$L_{2y}(\boldsymbol{k}) = \frac{1}{2}\{(cos\,\theta - 1)L'_{1x}(\boldsymbol{k}) + (cos\,\theta + 1)L'_{2x}(\boldsymbol{k}) - sin\,\theta\,(L'_{1z}(\boldsymbol{k}) + L'_{2z}(\boldsymbol{k}))\};$$
$$L_{3x}(\boldsymbol{k}) = \frac{1}{2}\{sin\,\theta\,(L'_{1x}(\boldsymbol{k}) + L'_{2x}(\boldsymbol{k})) + L'_{1z}(\boldsymbol{k})(cos\,\theta - 1) + L'_{2z}(\boldsymbol{k})(cos\,\theta + 1)\};$$
$$L_{3y}(\boldsymbol{k}) = \frac{1}{2}\{(cos\,\theta - 1)F'_x(\boldsymbol{k}) + (cos\,\theta + 1)L'_{3x}(\boldsymbol{k}) - sin\,\theta\,(F'_z(\boldsymbol{k}) + L'_{3z}(\boldsymbol{k}))\};$$
$$F_z(\boldsymbol{k}) = L'_{1y}(\boldsymbol{k}); \quad L_{1z}(\boldsymbol{k}) = F'_y(\boldsymbol{k}); \quad L_{2z}(\boldsymbol{k}) = L'_{3y}(\boldsymbol{k}); \quad L_{3z}(\boldsymbol{k}) = L'_{2y}(\boldsymbol{k});$$

To make the symmetry properties more transparent, we introduce the same linear combinations (Eq. S4.2) for the sublattice bosonic operators $a_\alpha(\boldsymbol{k})$, for $\alpha = 1,2,3,4$ (12):

$$a_F(\boldsymbol{k}) = \frac{1}{2}\{a_1(\boldsymbol{k}) + a_2(\boldsymbol{k}) + a_3(\boldsymbol{k}) + a_4(\boldsymbol{k})\};$$

$$a_{L_1}(\boldsymbol{k}) = \frac{1}{2}\{a_1(\boldsymbol{k}) + a_2(\boldsymbol{k}) - a_3(\boldsymbol{k}) - a_4(\boldsymbol{k})\};$$

$$a_{L_2}(\boldsymbol{k}) = \frac{1}{2}\{a_1(\boldsymbol{k}) - a_2(\boldsymbol{k}) + a_3(\boldsymbol{k}) - a_4(\boldsymbol{k})\}; \qquad \text{(Eq S4.8)}$$

$$a_{L_3}(\boldsymbol{k}) = \frac{1}{2}\{a_1(\boldsymbol{k}) - a_2(\boldsymbol{k}) - a_3(\boldsymbol{k}) + a_4(\boldsymbol{k})\}.$$

Next, we apply the Holstein-Primakoff transformation to convert the operators ***L'*** to the bosonic operators $a_L(\boldsymbol{k}),\ a_L^+(\boldsymbol{k})$ (*L= F, L*$_1$*, L*$_2$*, L*$_3$):

$$L'_x(\boldsymbol{k}) = \sqrt{4s}Q_L(\boldsymbol{k});\ L'_y(\boldsymbol{k}) = i\sqrt{4s}P_L(-\boldsymbol{k});$$
$$Q_L(\boldsymbol{k}) = \frac{1}{\sqrt{2}}\{a_L^+(-\boldsymbol{k}) + a_L(\boldsymbol{k})\};\ P_L(\boldsymbol{k}) = \frac{1}{\sqrt{2}}\{a_L^+(\boldsymbol{k}) - a_L(-\boldsymbol{k})\} \qquad \text{(Eq S4.9)}$$

The z' components of the operators ***L'*** can be expressed in terms of operators *Q* and *P* (see Eq.10 in ref *15*). With the help of the relations (Eq. S4.7) and (Eq. S4.9) we can thus obtain a spin-wave Hamiltonian that is the quadratic part of the Hamiltonian (Eq. S4.3) with respect to bosonic operators (Eq. S4.8):

$$\widehat{H}^{(2)} = \frac{1}{2}\sum_{k,\sigma}\{q_\sigma(\boldsymbol{k})Q_\sigma(\boldsymbol{k})Q_\sigma(-\boldsymbol{k}) - p_\sigma(\boldsymbol{k})P_\sigma(\boldsymbol{k})P_\sigma(-\boldsymbol{k})\} +$$
$$+\sum_k \begin{Bmatrix} q_{03}(\boldsymbol{k})Q_0(\boldsymbol{k})Q_3(-\boldsymbol{k}) - p_{03}(\boldsymbol{k})P_{03}(\boldsymbol{k})P_L(-\boldsymbol{k}) + \\ +q_{12}(\boldsymbol{k})Q_1(\boldsymbol{k})Q_2(-\boldsymbol{k}) - p_{12}(\boldsymbol{k})P_1(\boldsymbol{k})P_2(-\boldsymbol{k}) \end{Bmatrix} \qquad \text{(Eq. S4.10)}$$

Here the subscripts σ=0,1,2,3 correspond to σ=*F, L*$_1$*, L*$_2$*, L*$_3$. The coefficients $q_\sigma(\boldsymbol{k})$ and $p_\sigma(\boldsymbol{k})$ in the Hamiltonian (Eq. S4.10) are defined by the expressions:

$$q_0(\boldsymbol{k}) = 8S[a(0) + d(\boldsymbol{k})]; p_0(\boldsymbol{k}) = 8S[a(0) - A(\boldsymbol{k})];$$
$$q_1(\boldsymbol{k}) = 8S[a(0) - d(\boldsymbol{k})];\ p_1(\boldsymbol{k}) = 8S[a(0) + A(\boldsymbol{k})];$$
$$q_2(\boldsymbol{k}) = 8S[a(0) - b(\boldsymbol{k})]; p_2(\boldsymbol{k}) = 8S[a(0) - B(\boldsymbol{k})];$$
$$q_3(\boldsymbol{k}) = 8S[a(0) + b(\boldsymbol{k})];\ p_3(\boldsymbol{k}) = 8S[a(0) + B(\boldsymbol{k})]; \qquad \text{(Eq. S4.11)}$$
$$q_{12}(\boldsymbol{k}) = 4S[C(0) - c(\boldsymbol{k})];\quad p_{12}(\boldsymbol{k}) = 4S[C(0) + C(\boldsymbol{k})];$$
$$q_{03}(\boldsymbol{k}) = 4S[C(0) + c(\boldsymbol{k})];\quad p_{03}(\boldsymbol{k}) = 4S[C(0) - C(\boldsymbol{k})];$$

$$a(0) = [(A(0) - B(0))\cos\theta + (A(0) + B(0))(1 + \sin^2\theta)]/2;$$
$$d(\boldsymbol{k}) = [A(\boldsymbol{k})(1 + \cos\theta) + B(\boldsymbol{k})(1 - \cos\theta) - (A(\boldsymbol{k}) - B(\boldsymbol{k}) + C(\boldsymbol{k}))\sin^2\theta]/2;$$

$$b(\boldsymbol{k}) = [A(\boldsymbol{k})(1 - \cos\theta) + B(\boldsymbol{k})(1 + \cos\theta) - (A(\boldsymbol{k}) - B(\boldsymbol{k}) + C(\boldsymbol{k}))\sin^2\theta]/2;$$
$$c(\boldsymbol{k}) = C(\boldsymbol{k}) - (A(\boldsymbol{k}) - B(\boldsymbol{k}) + C(\boldsymbol{k}))\sin^2\theta\,;$$

The energy of the spin waves will be defined by the standard Bogolyubov transformation of the bosonic operators $a_L(\boldsymbol{k})$, $a_L^+(\boldsymbol{k})$ into the $\xi_\nu^+(\boldsymbol{k})$, $\xi_\nu(\boldsymbol{k})$ operators of the creation and annihilation a magnon of the νth branch:

$$Q_L(\boldsymbol{k}) = \frac{1}{\sqrt{2}}\sum_\nu t_{L\nu}(\boldsymbol{k})\{\xi_\nu^+(-\boldsymbol{k}) + \xi_\nu(\boldsymbol{k})\}$$
$$P_L(\boldsymbol{k}) = \frac{1}{\sqrt{2}}\sum_\nu d_{L\nu}(\boldsymbol{k})\{\xi_\nu^+(\boldsymbol{k}) - \xi_\nu(-\boldsymbol{k})\} \quad \text{(Eq. S4.12)}$$

The coefficients *t* and *d* satisfy the usual normalization conditions.

$$\sum_\nu \{t_{L\nu}(\boldsymbol{k})d^*_{L'\nu}(\boldsymbol{k}) + t^*_{L'\nu}(\boldsymbol{k})d_{L\nu}(\boldsymbol{k})\} = 2\delta_{LL'}$$
$$\sum_L \{t_{L\nu}(\boldsymbol{k})d^*_{L\nu'}(\boldsymbol{k}) + t^*_{L\nu'}(\boldsymbol{k})d_{L\nu}(\boldsymbol{k})\} = 2\delta_{\nu\nu'} \quad \text{(Eq. S4.13)}$$

The pairs of acoustic modes and the pairs of optical modes are degenerate within the accuracy of the exchange approximation.

The spin wave energies and the *t-d* coefficients of bulk YBCO ($J_{MC} = 0; J_\parallel^{IF} = J_\parallel; \theta = 0;$) have the simple form:

$$t_{L\nu}(\boldsymbol{k}) = \{\frac{p_L(\boldsymbol{k})}{q_L(\boldsymbol{k})}\}^{\frac{1}{4}}; \quad d_{L\nu}(\boldsymbol{k}) = \{\frac{q_L(\boldsymbol{k})}{p_L(\boldsymbol{k})}\}^{\frac{1}{4}};$$
$$\varepsilon_{AM1}(\boldsymbol{k}) = \sqrt{p_0(\boldsymbol{k})q_0(\boldsymbol{k})}; \qquad \varepsilon_{AM2}(\boldsymbol{k}) = \sqrt{p_1(\boldsymbol{k})q_1(\boldsymbol{k})}; \quad \text{(Eq. S4.14)}$$
$$\varepsilon_{OM1}(\boldsymbol{k}) = \sqrt{p_2(\boldsymbol{k})q_2(\boldsymbol{k})}; \qquad \varepsilon_{OM2}(\boldsymbol{k}) = \sqrt{p_3(\boldsymbol{k})q_3(\boldsymbol{k})};$$

The above-mentioned divergence of the acoustic spin wave fluctuations is evident from the coefficients $d_{0AM1}(\boldsymbol{k}) = \{\frac{q_0(\boldsymbol{k})}{p_0(\boldsymbol{k})}\}^{\frac{1}{4}}$; $t_{1AM2}(\boldsymbol{k}) = \{\frac{p_1(\boldsymbol{k})}{q_1(\boldsymbol{k})}\}^{\frac{1}{4}}$; for which $\lim_{k\to 0} p_0(\boldsymbol{k}) = 0$; $\lim_{k\to 0} q_1(\boldsymbol{k}) = 0$. The relations (Eq. S4.14) for magnon energies can be used to estimate the magnitude of the intraplane exchange interaction of the bulk-like CuO_2 layer from the M2 mode that governs the RIXS data at 931 eV.

The fitting, shown in Fig. 5A and 5B in the main manuscript yields a value of $J_{||}$ = 130 meV that is typical for bulk YBCO(*11*, *14*). Here we have used a fixed value of $J_\perp$ = 7 meV that also agrees well with bulk-like properties.

The last term of the summation in the Hamiltonian in equation Eq. S4.10 represents a set of mixing terms between two pairs of acoustic and optic modes that arises from the inequality of the interfacial and bulk-like CuO_2 planes that is caused by the reduction of the intraplanar exchange $\delta J_{\parallel} = J_{\parallel} - J_{\parallel}^{IF}$. Note that there exists no corresponding mixing between the pairs of acoustic modes and between the pairs of optical modes which thus remain double degenerated in $\boldsymbol{k}$-space. The respective solutions for the spin wave energies and the t-d coefficients in our double layer model can be found analytically. We apply these solutions to determine the value of $\delta J_{\parallel} = J_{\parallel} - J_{\parallel}^{IF}$ from a fit to the M1 mode in the RIXS data at 930.5 eV as shown in Fig. 5A and 5B.

From the above-described fitting we obtain an unusually large suppression of the interfacial in-plane AFM exchange of $\delta J_{\parallel} = J_{\parallel} - J_{\parallel}^{IF} = 60$ meV to a value of $J_{\parallel}^{IF} = 70$ meV. The energy splitting arises from the mixing between the acoustic and optical spin waves which increases towards larger $\boldsymbol{k}$-vectors and becomes maximal at the Brillouin zone boundary. We recall that this strong reduction of the energy of the interfacial magnon mode (M1-mode) can be naturally explained in terms of the orbital reconstruction of interface copper ions which leads to a mixed $d_{x^2-y^2}$ and $d_{3z^2-r^2}$ orbital character of the holes.

S4.3. Intensity of spin waves in the RIXS spectra of YBCO/NCSMO multilayers

According to Ref. *(16,17)* the RIXS intensity of the spin waves of the ν-th branch in cross-polarization is defined by the square of the matrix element corresponding to the transition between the states with a difference in magnon numbers $n_\nu(\boldsymbol{k})$ equal to unity:

$$M_{k\nu} \propto \langle n_\nu(\boldsymbol{k}) + 1 | (\boldsymbol{\sigma}_{out} \times \boldsymbol{\pi}_{in}) \cdot \{ \sum_{\alpha=1,2,3,4} f_\alpha(\boldsymbol{Q}) e^{-i2\pi \boldsymbol{Q}\boldsymbol{\rho}_\alpha} \boldsymbol{S}_\alpha(\boldsymbol{k}) \} | n_\nu(\boldsymbol{k}) \rangle \qquad \text{(Eq. S4.15)}$$

Here $\boldsymbol{\pi}_{in}, \boldsymbol{\sigma}_{out}$ denote the polarizations of the incoming and outgoing x-ray beams, $\boldsymbol{Q}$ is the scattering vector, $f_\alpha(\boldsymbol{Q})$ the form-factor of the copper ion α, and $\boldsymbol{\rho}_\alpha$ the position of copper ion α in the primitive magnetic cell. For the bulk-like YBCO layers equation (Eq. S4.15) can be expressed in terms of operators $\boldsymbol{L}$ using the relation (Eq. S4.2).

$$M_{k\nu} \propto \langle n_\nu(\boldsymbol{k}) + 1 | (\boldsymbol{\sigma_{out}} \times \boldsymbol{\pi_{in}}) \cdot \{ f(\boldsymbol{Q}) \sum_{L=0,1,2,3} G_L(\boldsymbol{Q}) \boldsymbol{L}(\boldsymbol{k}) \} | n_\nu(\boldsymbol{k}) \rangle; \qquad \text{(Eq. S4.16)}$$

Here it is supposed that all copper ions are in the same orbital state and therefore have the same form-factor, $f(\boldsymbol{Q})$. The $\boldsymbol{Q}$ – dependence of the form-factor for the copper ions at the Cu L-edge can be derived in the frame of a single ion model described in Ref. *(18)* assuming, for example, that all the holes are in a $d_{x^2-y^2}$ orbital ground state. This calculation considers the atomic symmetries of the core-level $2p$ and valence $3d$-orbitals

and the geometry of the experimental setup, namely π−polarized incident light and a scattering angle of 50°. The matrix elements for elastic scattering are therefore obtained as the product of two dipole transitions: between the core-level state $2p$ to a valence state $3d$ and de-excitation from the same $3d$ state back to the $2p$ state. Selection rules involving the addition of angular momenta are computed using the Wigner 3-j symbols.

As we are interested in the intensity of magnetic excitations close to the elastic line, we calculate cross-sections involving a spin-flip with a spin initially along the $\boldsymbol{a}$ direction for either $d_{x^2-y^2}$ or $d_{3z^2-r^2}$ ground state orbitals (but not involving any change of orbital character in the *3d* valence state).

Fig S8. summarizes the results for the single ion-model calculation. Please note that any linear combination of the data in Fig S8(a) and S8(b) cannot reproduce the experimental trend. It indulges us to account for the $t-d$ coefficients.

The geometrical (or structural) factors $G_L(\boldsymbol{Q})$ are analogous to those for inelastic neutron scattering on spin waves *(15)*:

$$G_0(\boldsymbol{Q}) = cos\frac{\pi}{2}\boldsymbol{Q}(\boldsymbol{A}+\boldsymbol{B})\, cos\, \pi\, \boldsymbol{Q}_\perp \boldsymbol{d}; \quad G_1(\boldsymbol{Q}) = -\, sin\frac{\pi}{2}\boldsymbol{Q}(\boldsymbol{A}+\boldsymbol{B})\, sin\, \pi\, \boldsymbol{Q}_\perp \boldsymbol{d}$$

$$G_2(\boldsymbol{Q}) = i\, sin\frac{\pi}{2}\boldsymbol{Q}(\boldsymbol{A}+\boldsymbol{B})\, cos\, \pi\, \boldsymbol{Q}_\perp \boldsymbol{d}; \quad G_3(\boldsymbol{Q}) = i\, cos\frac{\pi}{2}\boldsymbol{Q}(\boldsymbol{A}+\boldsymbol{B})\, sin\, \pi\, \boldsymbol{Q}_\perp \boldsymbol{d}; \qquad \text{(Eq. 4.17)}$$

Here we use a bulk-like value for the distance between copper ions along the z-axis of d = 3.388Å. Finally, the $\boldsymbol{Q}$-dependence of the RIXS magnon cross-section can be expressed in terms of the $\boldsymbol{\sigma}_{out}$, $\boldsymbol{\pi}_{in}$-polarization, the form factor $f(\boldsymbol{Q})$, the structural factor $G(\boldsymbol{Q})$ and the *t-d* coefficients of the spin – waves.

For the resonance condition of the interfacial CuO_2 layer at 930.5 eV, for which mainly the interfacial copper ions Cu1 and Cu4 ions participate in the scattering process, we assume that the contributions of the Cu2 and Cu3 ions (from the other CuO_2 layer of the bilayer unit that is located further away from the interface) can be omitted. The respective matrix element for the RIXS process thus has the following form.

$$M_{k\nu} \propto \langle n_\nu(\boldsymbol{k}) + 1 | (\boldsymbol{\sigma_{out}} \times \boldsymbol{\pi_{in}}) \cdot f(\boldsymbol{Q}) \cdot \{ e^{-i2\pi \boldsymbol{Q\rho_1}} \boldsymbol{S}_1(\boldsymbol{k}) + e^{-i2\pi \boldsymbol{Q\rho_4}} \boldsymbol{S}_4(\boldsymbol{k}) \} | n_\nu(\boldsymbol{k}) \rangle \qquad \text{(Eq. 4.18)}$$

Using the relation (Eq. S4.2) we thus can rewrite the equation (Eq. S4.18) for the interfacial CuO_2 layers in terms of the following form of the $\boldsymbol{L}$ operators:

$$M_{kv} \propto \frac{1}{2}\langle n_v(\boldsymbol{k})+1|(\boldsymbol{\sigma_{out}} \times \quad \boldsymbol{\pi_{in}}) \cdot f(\boldsymbol{Q}) \cdot \{cos\frac{\pi}{2}\boldsymbol{Q}(\boldsymbol{A}+\boldsymbol{B})[\boldsymbol{F}(\boldsymbol{k}) + \boldsymbol{L}_3(\boldsymbol{k})] + i\, sin\frac{\pi}{2}\boldsymbol{Q}(\boldsymbol{A}+\boldsymbol{B})[\boldsymbol{L}_1(\boldsymbol{k})+\boldsymbol{L}_2(\boldsymbol{k})]\}|n_v(\boldsymbol{k})\rangle \quad \text{(Eq. S4.19)}$$

In this simplified case, the scattering occurs only from the interfacial CuO_2 layer, the contribution of $\boldsymbol{Q}_\perp$ to the structural factor thus is absent. Furthermore, we demonstrate that the doubly degenerate acoustic modes dominate the scattering from the magnons in the interfacial CuO_2 layer, whereas the contribution from the optical magnon modes can be neglected.

It is important to note a rather general similarity between Eq. S4.19 and Eq. S4.16 with respect to a divergence of the acoustic spin wave fluctuations at $\boldsymbol{k}\rightarrow 0$ ascribed by operators L_{1y} and L_{1z} that is in both cases fully suppressed by the structural factors. However, theoretically the divergence of the RIXS magnon cross section can occur at the $\boldsymbol{Q}_{||} = \boldsymbol{A}^*$ (at so called (π, π) -point, the center of the next magnetic Brillouin zone) which can be observed if $Q_\perp \neq 0$ for the scattering on the bulk (*17*) and under absence of such a restriction for the scattering on the interfacial plane.

The $\boldsymbol{Q}$ – dependence of the RIXS intensity as calculated for the bulk-like CuO_2 planes, with the help of Eq. S4.16, and for the interfacial CuO_2 layer, with the help of Eq. S4.19 is shown by the solid lines in Figs. 5C-5F of the manuscript. The calculated intensity contains contributions of a sum of independent contributions for the four spin wave branches (i.e., from two doubly degenerate optic modes and the two doubly degenerate acoustic modes). For the bulk like magnon mode (M2 mode) there is reasonable agreement in Figs. 5C and 5D between the measured RIXS data and the prediction of our minimal spin-wave theory. To the contrary, for the M1 mode due to the magnons in the interfacial CuO_2 layers, there a clear discrepancy between the $\boldsymbol{Q}$-dependence of the measured values and the theoretical prediction. Notably, the former exhibit a steep increase of the scattering intensity towards small k-values whereas the latter predict a strong decrease.

The suppression of the contribution of the highly intensive acoustic spin-wave fluctuations to the inelastic scattering process at $\boldsymbol{k}$ vectors close to the Γ – point has a very general origin. Indeed, the acoustic low energy modes induce a rotation of the main order parameter that involves spin fluctuations of equal magnitude but of opposite directions on the nearest neighbor sites. Therefore, irrespective of the number of AFM sublattices, the scattering cross-section of acoustic spin-waves is strongly suppressed and vanishes in the limit of $\boldsymbol{k}\rightarrow 0$, due to the mutual cancelation of in-phase and out-of-phase fluctuations with the same scattering amplitude at the neighboring up- and down- spin sites. This destructive-interference-effect manifests itself as an action of the structural factor that is proportional to $\boldsymbol{k}$, as is evident from Eq. S4.16 and Eq. S4.19.

The obvious way to overcome this destructive interference effect on the magnon intensity towards small wave vectors is a mechanism that gives rise to different scattering amplitudes of the neighboring Cu ions. A natural candidate in the context of the cuprate/manganite interface is a spatial alternation of the orbital character of the holes of the interfacial Cu ions. The simplest model that we consider is the checkerboard type of the $d_{x^2-y^2}$ and $d_{3z^2-r^2}$ orbital order shown in Fig. 6A of the main manuscript which has the same translation symmetry as the underlying AF spin order. Note, that this orbital order also implies a strong decrease of the interfacial intraplane exchange. The resulting scattering amplitude of the (M1) magnon excitations in the RIXS spectra has the form:

$$M_{kv} \propto \langle n_v(\boldsymbol{k})+1|(\boldsymbol{\sigma_{out}}\times\ \boldsymbol{\pi_{in}})\cdot\{f(\boldsymbol{Q},x^2)e^{-i2\pi\boldsymbol{Q}\rho_1}\boldsymbol{S}_1(\boldsymbol{k}) + f(\boldsymbol{Q},z^2)e^{-i2\pi\boldsymbol{Q}\rho_4}\boldsymbol{S}_4(\boldsymbol{k})\}|n_v(\boldsymbol{k})\rangle \quad \text{(Eq. S4.20)}$$

Here we introduce the different form-factors $f(\boldsymbol{Q},x^2),\ \ f(\boldsymbol{Q},z^2)$ for the copper ions in the $d_{x^2-y^2},\ \ d_{3z^2-r^2}$ orbital states, respectively. The different orbital states provide different scattering amplitudes through the different amplitudes of the form-factor. After respective substitutions of the sublattices spin operators $\boldsymbol{S}$ into the operators $\boldsymbol{L}$ we thus obtain:

$$\begin{aligned}M_{kv} \propto \tfrac{1}{2}\langle n_v(\boldsymbol{k})+1|(\boldsymbol{\sigma_{out}}\times\ \boldsymbol{\pi_{in}})\cdot \\ \{[f(\boldsymbol{Q},x^2)+f(\boldsymbol{Q},z^2)][cos\tfrac{\pi}{2}\boldsymbol{Q}(\boldsymbol{A}+\boldsymbol{B})[\boldsymbol{F}(\boldsymbol{k})+\boldsymbol{L}_3(\boldsymbol{k})]+i\,sin\tfrac{\pi}{2}\boldsymbol{Q}(\boldsymbol{A}+\boldsymbol{B})[\boldsymbol{L}_1(\boldsymbol{k})+\boldsymbol{L}_2(\boldsymbol{k})]] + \\ +[f(\boldsymbol{Q},x^2)-f(\boldsymbol{Q},z^2)][cos\tfrac{\pi}{2}\boldsymbol{Q}(\boldsymbol{A}+\boldsymbol{B})[\boldsymbol{L}_1(\boldsymbol{k})+\boldsymbol{L}_2(\boldsymbol{k})]+i\,sin\tfrac{\pi}{2}\boldsymbol{Q}(\boldsymbol{A}+\boldsymbol{B})[\boldsymbol{F}(\boldsymbol{k})+\boldsymbol{L}_3(\boldsymbol{k})]]\}|n_v(\boldsymbol{k})\rangle\end{aligned} \quad \text{(Eq. S4.21)}$$

The first term in the braces represents the result described above in Eq. S4.19. Here, at low $\boldsymbol{k}$, the divergent contributions from the operators L_{1y} and L_{1z}, are fully suppressed by the structure factor $sin\frac{\pi}{2}\boldsymbol{Q}(\boldsymbol{A}+\boldsymbol{B})$.

In contrast, for the second term in the braces, which is proportional to the form-factor difference $f(\boldsymbol{Q},x^2)-f(\boldsymbol{Q},z^2)$, the contribution of the strong spin-wave fluctuations of the magnetic order parameter does not get suppressed toward small wave vectors. It rather gives rise to a contribution of the acoustic magnons of the interfacial CuO_2 layers that strongly increases towards small wave vectors and thus can explain the observed strong increase of the M1 mode intensity in the RIXS spectra, as shown by the solid line in Fig. 6B in the main manuscript. Here the different form factors for the spin with $d_{x^2-y^2}$ and $d_{3z^2-r^2}$ orbital character have been calculated with the single ion model described in

Ref.(18). Note that the twinning of the orthorhombic structure of YBCO has been considered by averaging their contributions to the RIXS cross-section.

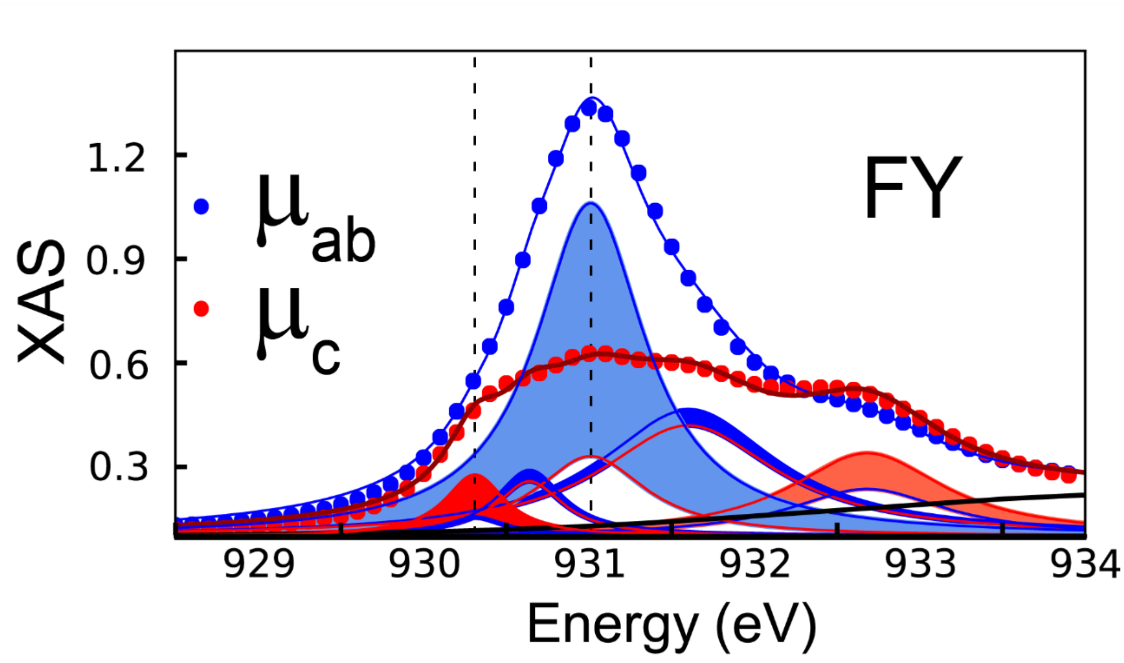

Fig. S1. FY spectra and multi-peak fit for the in-plane and out-of-plane components of the linear polarized incident x-rays. Blue (red) shaded areas denote contributions for which $\boldsymbol{\mu_{ab}} - \boldsymbol{\mu_c}$ >0. (<0).

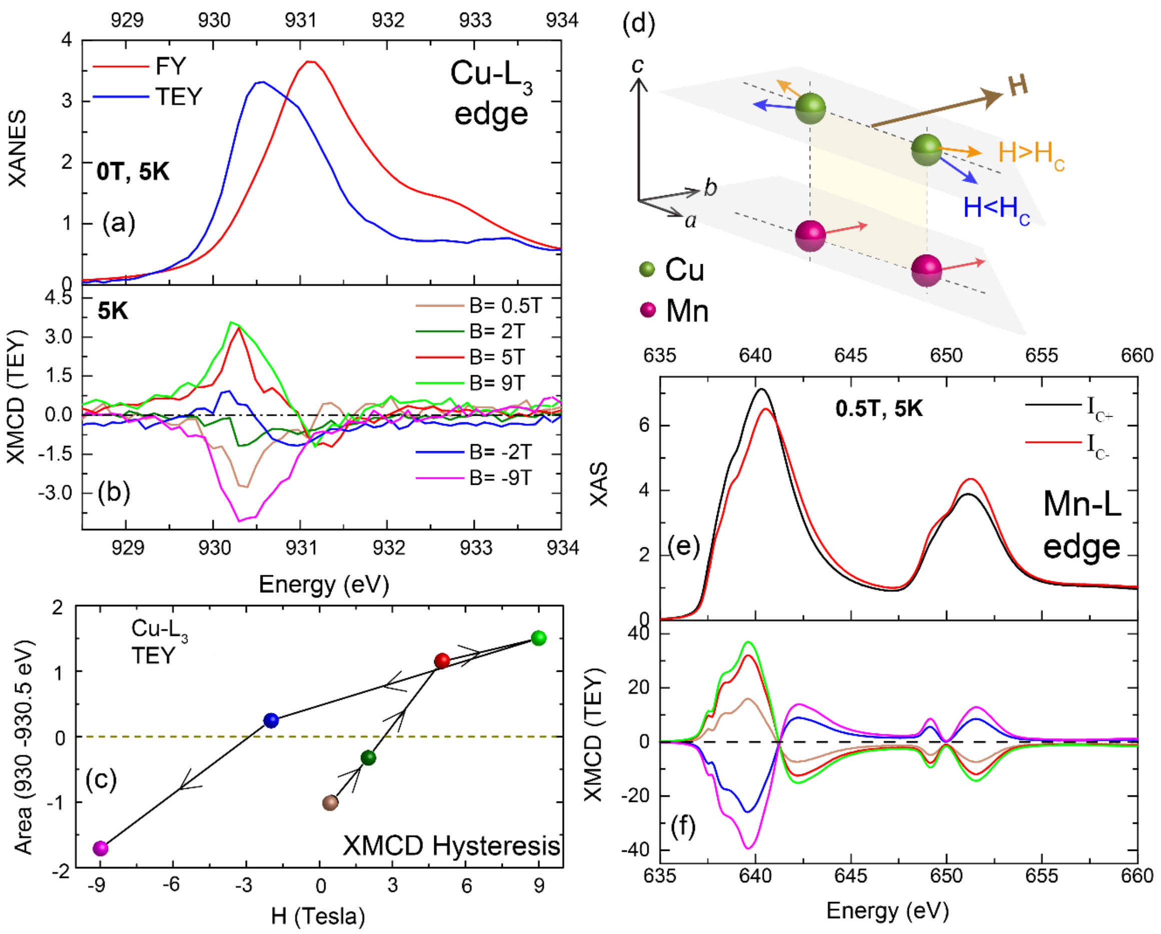

Fig. S2. (a) XAS spectra in FY and TEY modes that reveal the distinct resonances of the bulk-like and interfacial Cu ions around 930.4 and 931 eV, respectively. (b) Corresponding Cu-XMCD spectra in TEY mode at different applied fields. They confirm that the magnetic signal is maximal around the resonance of the interfacial Cu ions at 930.4 eV and that it undergoes a sign change around 3 − 4 Tesla that is characteristic of a field-induced reorientation. (c) Magnetic field-loop of the integrated Cu-XCMD signal in panel (b). (d) Sketch of the mutual orientation of the ferromagnetic Mn moment and the induced Cu moment, due to the canting of the AF Cu-spins, below (blue) and above (orange) the switching field, H_c. (e) Circularly polarized Mn-XAS spectra in TEY mode. (f) Corresponding Mn-XMCD spectra at different magnetic fields which confirm that the Mn moment is always parallel to the applied field.

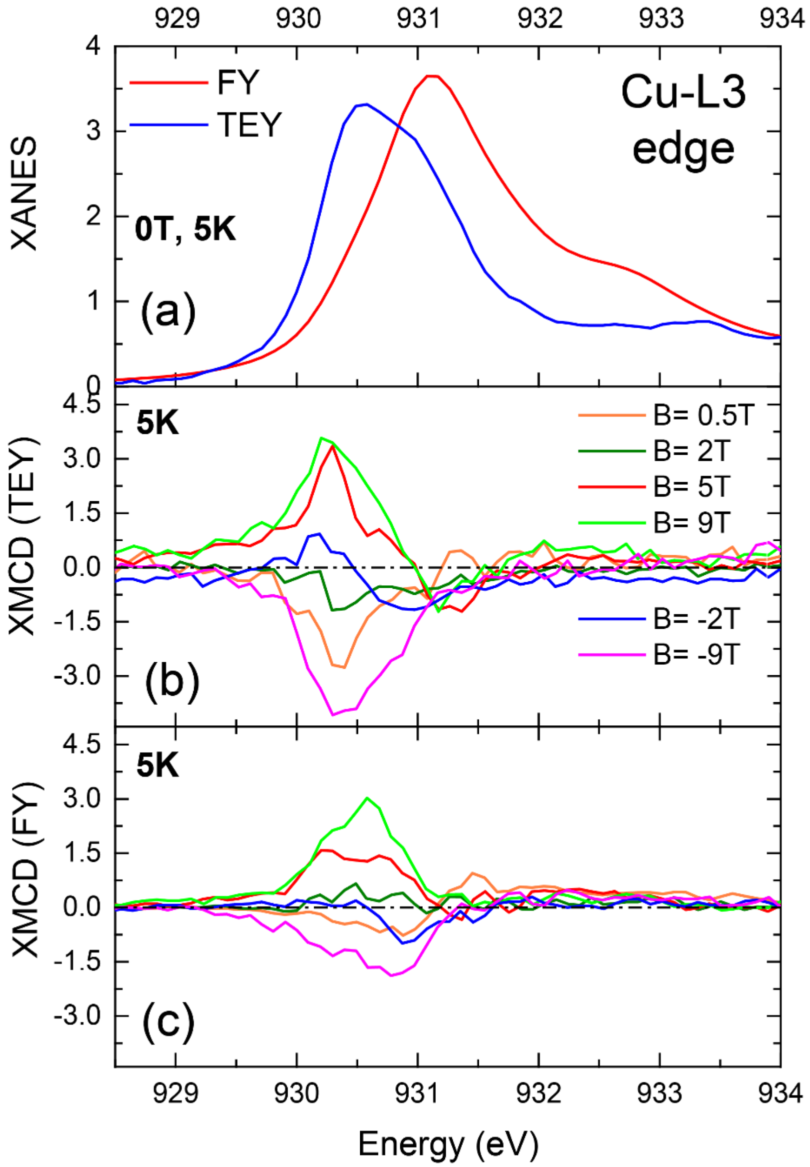

Fig. S3. Comparison of the XMCD in FY and TEY spectra at Cu-L3 edge.

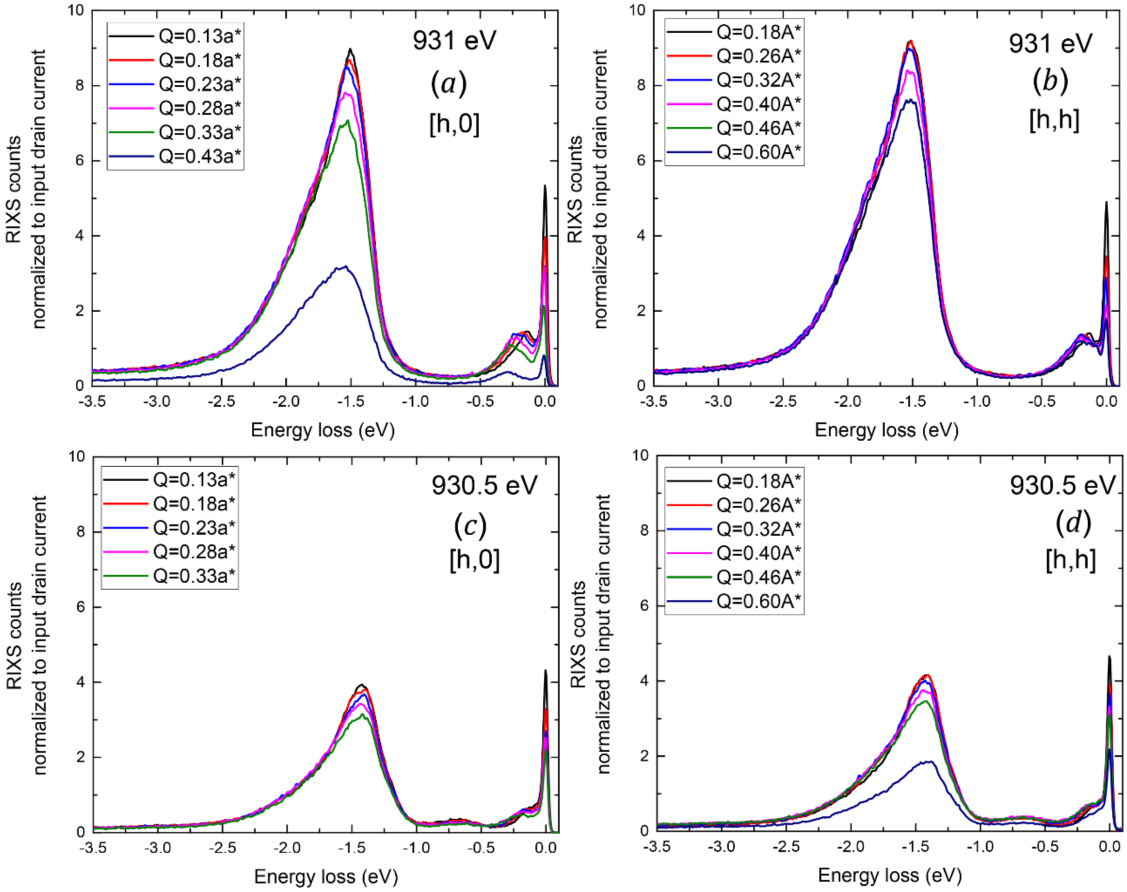

Fig S4. Cu-L_3 edge RIXS counts obtained by normalizing the signal to the input x-ray flux only: (a) and (b): RIXS spectra taken at the resonance of the bulk-like Cu ions at 931 eV along [h,0] and [h,h], respectively. (c) and (d): Corresponding RIXS spectra taken at the resonance of the interfacial Cu ions at 930.5 eV. The data taken at the largest value of Q|| show an intensity decrease that arise from a larger footprint of the beam at grazing incidence.

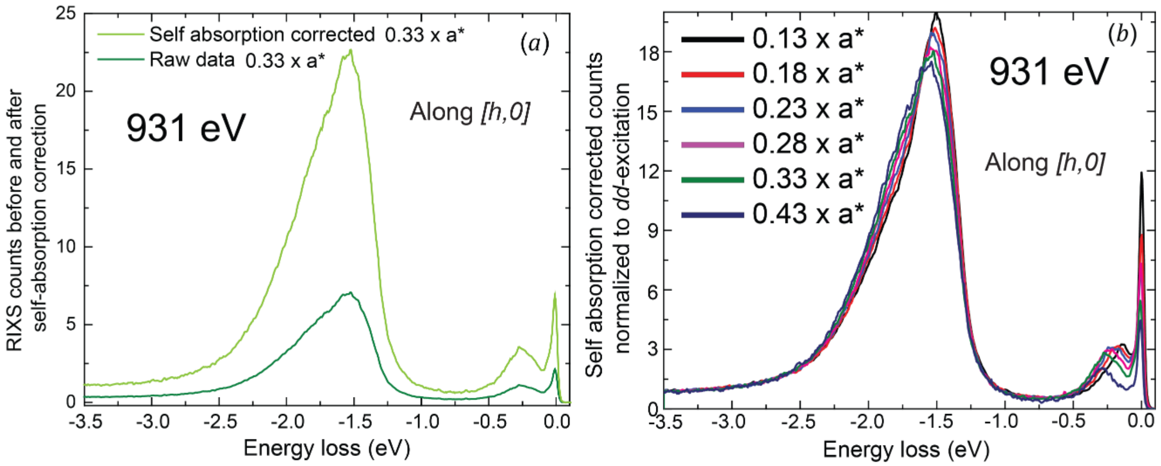

Fig S5. (a) Effect of self-absorption correction for a RIXS spectrum along [h,0] at 0.33a*. (b) Data normalization to the dd-excitation for the footprint correction of the RIXS spectra along [h,0] at 931 eV.

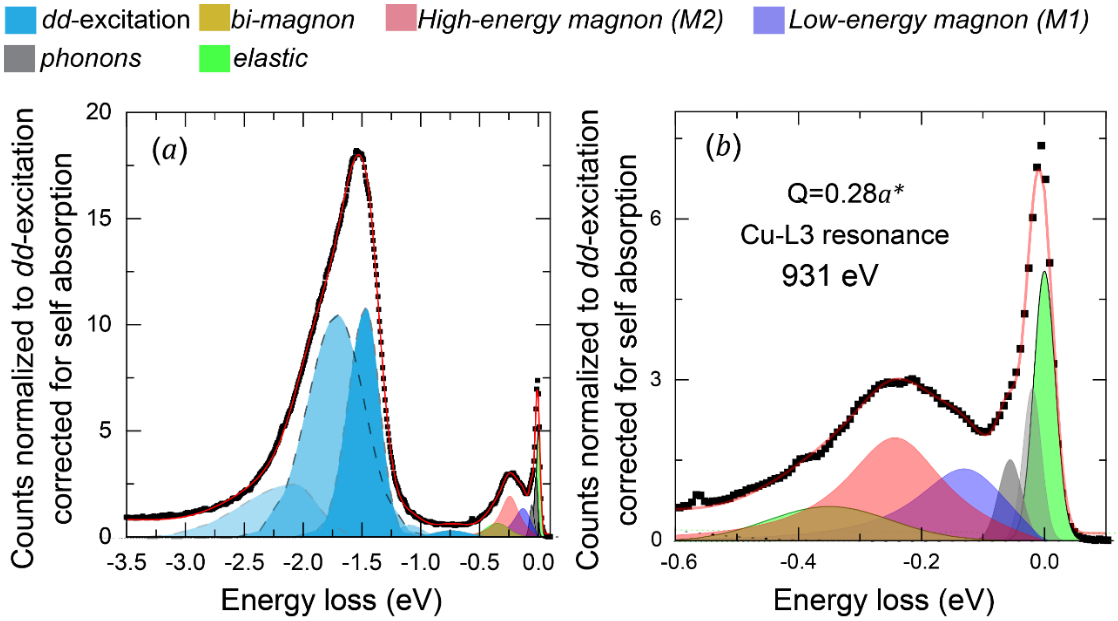

Fig S6. A model fit of a self-absorption corrected and normalized spectrum at 931 eV at 0.28a* along [h,0]. The various shaded and colored areas show the different contributions of the lattice, electronic and magnetic excitations.

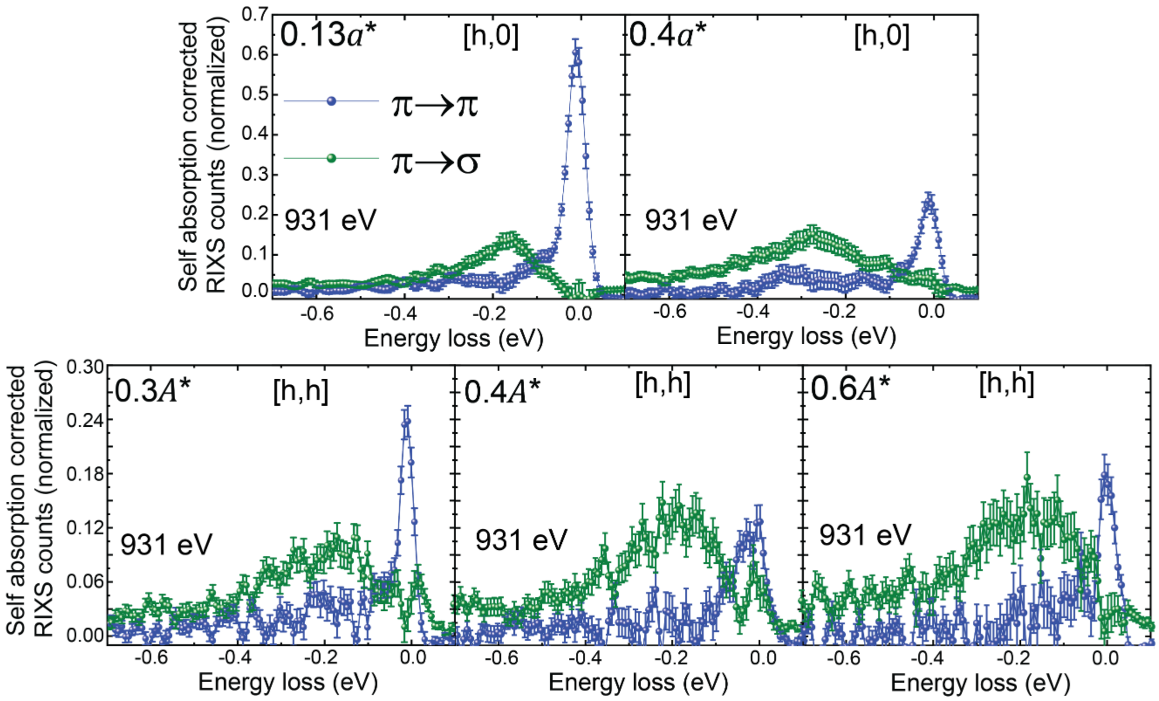

Fig S7. Results of all RIXS polarimetry analysis of the scattered beam: Note that the spin-flip component of the scattering dominates the neighborhood of M1 and M2 modes for all spectra. Directions and coordinates in the BZ have been indicated

.

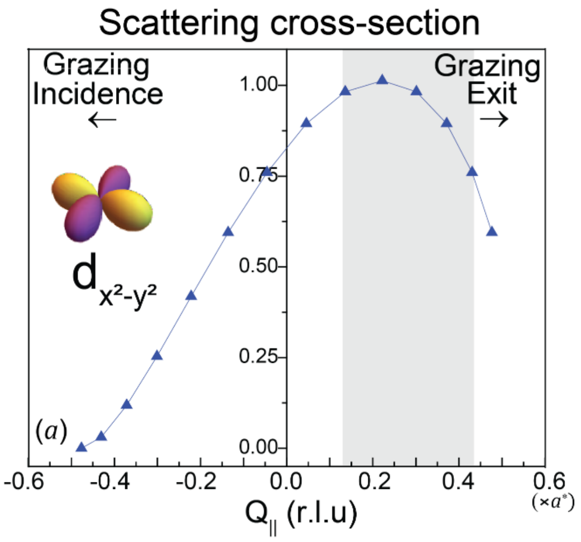

Fig S8. Summary of single ion model calculation for spin-flip scattering cross-section: (a) for $\boldsymbol{d_{x^2-y^2}}$ and (b) $\boldsymbol{d_{3z^2-r^2}}$ orbitals; the shaded region indicates the range of $\boldsymbol{Q}_{||}$ probed in our experiment along a*.

References:

1. Stohr J & Siegmann HC (2006) *Magnetism From Fundamentals to Nanoscale Dynamics* (Springer-Verlag Berlin Heidelberg, New York).

2. Andersen NH & Uimin G (1997) Model for the low-temperature magnetic phases observed in doped $YBa_2Cu_3O_{6+x}$. *Phys. Rev. B* 56:10840.

3. Sen K*, et al.* (2016) X-ray absorption study of the ferromagnetic Cu moment at the $YBa_2Cu_3O_7/La_{2/3}Ca_{1/3}MnO_3$ interface and variation of its exchange interaction with the Mn moment. *Phys. Rev. B* 93(205131):205131.

4. A. Alberca*, et al.* (2015) Element-specific magnetization redistribution at $YBa_2Cu_3O_7/La_{2/3}Ca_{1/3}MnO_3$ interfaces. *Phys. Rev. B* 92(174415):174415.

5. Gaina R*, et al.* (2021) Magnetic field dependence of the copper charge density wave order in a $YBa_2Cu_3O_7/Nd_{0.65}(Ca_{0.7}Sr_{0.3})_{0.35}MnO_3$ superlattice. *Phys. Rev. B* 104(174513):174513.

6. Minola M*, et al.* (2015) Collective Nature of Spin Excitations in Superconducting Cuprates Probed by Resonant Inelastic X-Ray Scattering. *Phys. Rev. Lett.* 114(217003):217003.

7. Robarts HC*, et al.* (2019) Anisotropic damping and wave vector dependent susceptibility of the spin fluctuations in $La_{2-x}Sr_xCuO_4$ studied by resonant inelastic x-ray scattering. *Phys. Rev. B* 100(214510):214510.

8. Magnuson M, Schmitt T, & Duda L-C (2018) Polarization-dependent resonant inelastic X-ray scattering study at the Cu L and O K-edges of $YBa_2Cu_3O_{7-x}$. *Journal of Electron Spectroscopy and Related Phenomena* 224(38):38-44.

9. Braicovich L*, et al.* (2020) Determining the electron-phonon coupling in superconducting cuprates by resonant inelastic x-ray scattering: Methods and results on $Nd_{1+x}Ba_{2-x}Cu_3O_{7-\delta}$. *Phys. Rev. Research* 2(023231):023231.

10. Lamsal J & Montfrooij W (2016) Extracting paramagnon excitations from resonant inelastic x-ray scattering experiments. *Phys. Rev. B* 93(214513):214513.

11. Tacon ML*, et al.* (2011) Intense paramagnon excitations in a large family of high-temperature superconductors. *Nature Physics* 7(725):725-73.

12. Chakhalian J *et al.,* (2006) Magnetism at the interface between ferromagnetic and superconducting oxides Nat. Phys., 2, 244.

13. Williams A *et al.,* (1988) Joint x-ray and neutron refinement of the structure of superconducting YBa2Cu3O7−x: Precision structure, anisotropic thermal parameters, strain, and cation disorder Phys. Rev. B 37, 7960(R) (1988)

14. Tranquada JM, G. Shirane, B. Keimer, S. Shamoto, & Sato M (1989) Neutron scattering study of magnetic excitations in $YBa_2Cu_3O_{6+x}$. *Phys. Rev. B* 40(4503):4503.

15. Sobolev VL*, et al.* (1994) Spin-wave spectrum and inelastic neutron scattering by magnons in Nd_2CuO_4. *Phys. Rev. B* 49:1170.

16. Haverkort MW (2010) Theory of Resonant Inelastic X-Ray Scattering by Collective Magnetic Excitations. *phys. Rev. Lett.* 105(167404):167404.

17. Ament LJP, Ghiringhelli G, Sala MM, Braicovich L, & Brink Jvd (2009) Theoretical Demonstration of How the Dispersion of Magnetic Excitations in Cuprate Compounds can be Determined Using Resonant Inelastic X-Ray Scattering. *Phys. Rev. Lett.* 103(117003):117003.

18. Moretti Sala M.*, et al.* (2011) Energy and symmetry of dd excitations in undoped layered cuprates measured by Cu L_3 resonant inelastic x-ray scattering. *New Journal of Physics* 13(043026):043026.
